# Supplementary material for: Three-Dimensional Genomic Structure and Cohesin Occupancy Correlate with Transcriptional Activity during Spermatogenesis
Source: Cell Rep. 2019 Jul 9;28(2):352–367.e9. doi: 10.1016/j.celrep.2019.06.037 (PMC6635386; doi:10.1016/j.celrep.2019.06.037)
Supplement: Document S1. Figures S1–S6 [file mmc1.pdf]

Cell Reports, Volume *28*

Supplemental Information

Three-Dimensional Genomic Structure and Cohesin Occupancy Correlate with Transcriptional Activity during Spermatogenesis

Covadonga Vara, Andreu Paytuví-Gallart, Yasmina Cuartero, François Le Dily, Francisca Garcia, Judit Salvà-Castro, Laura Gómez-H, Eva Julià, Catia Moutinho, Riccardo Aiese Cigliano, Walter Sanseverino, Oscar Fornas, Alberto M. Pendás, Holger Heyn, Paul D. Waters, Marc A. Marti-Renom, and Aurora Ruiz-Herrera

Figure S1

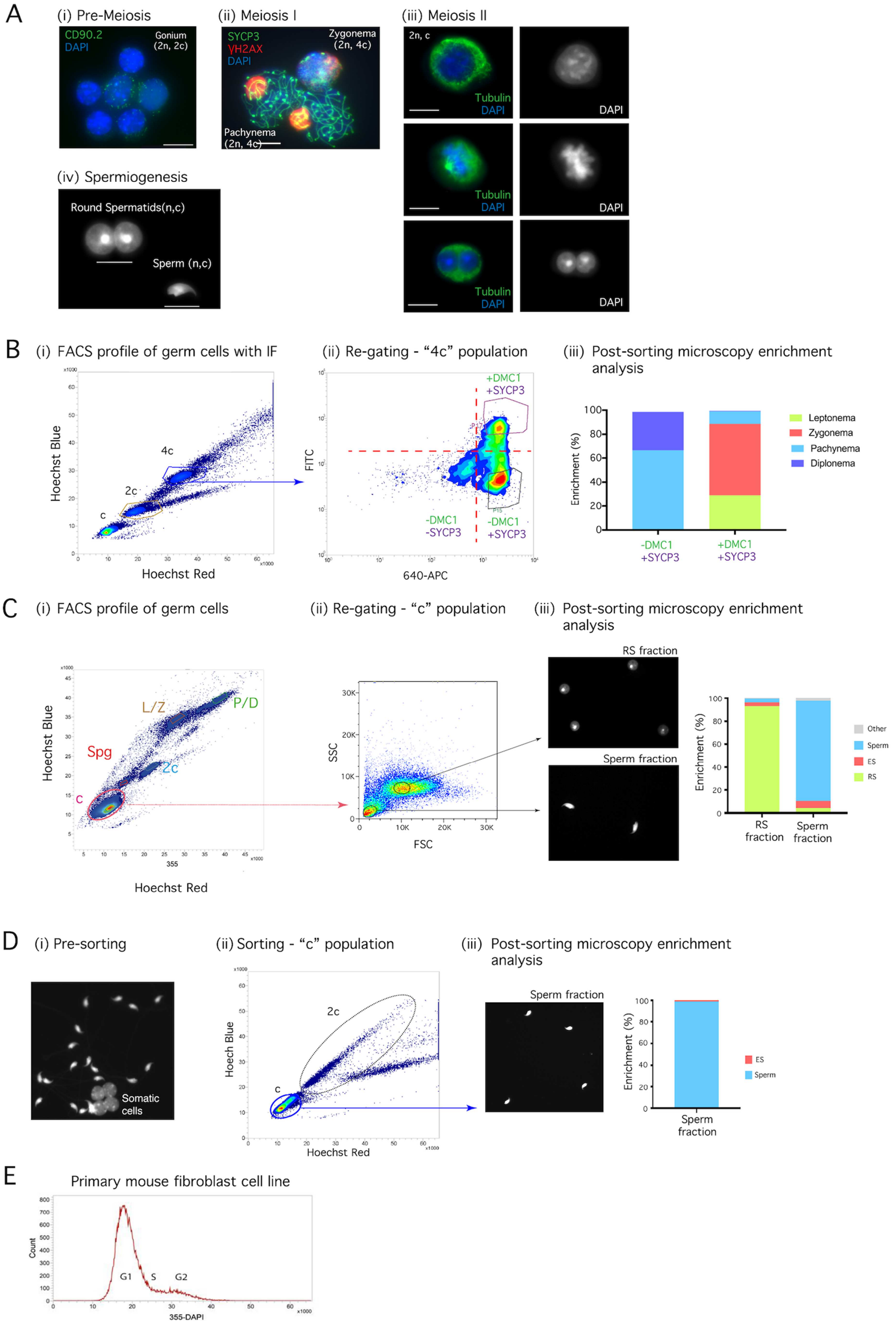
A
(i) Pre-Meiosis
CD90.2
DAPI
Gonium (2n, 2c)
(ii) Meiosis I
SYCP3
γH2AX
DAPI
Zygonema (2n, 4c)
Pachynema (2n, 4c)
(iii) Meiosis II
2n, c
Tubulin
DAPI
(iv) Spermiogenesis
Round Spermatids(n,c)
Sperm (n,c)
B
(i) FACS profile of germ cells with IF
Hoechst Blue
Hoechst Red
4c
2c
c
(ii) Re-gating - "4c" population
FITC
640-APC
+DMC1 +SYCP3
-DMC1 -SYCP3
-DMC1 +SYCP3
(iii) Post-sorting microscopy enrichment analysis
Enrichment (%)
Leptonema
Zygonema
Pachynema
Diplonema
-DMC1 +SYCP3
+DMC1 +SYCP3
C
(i) FACS profile of germ cells
Hoechst Blue
Hoechst Red
L/Z
P/D
Spg
2c
c
(ii) Re-gating - "c" population
SSC
FSC
(iii) Post-sorting microscopy enrichment analysis
RS fraction
Sperm fraction
Enrichment (%)
Other
Sperm
ES
RS
D
(i) Pre-sorting
Somatic cells
(ii) Sorting - "c" population
Hoech Blue
Hoechst Red
2c
c
(iii) Post-sorting microscopy enrichment analysis
Sperm fraction
Enrichment (%)
ES
Sperm
E
Primary mouse fibroblast cell line
Count
G1
S
G2
355-DAPI

Figure S1: FACS strategies for isolation of highly enriched germ cell populations, related to Figure 1. (A) Representative immunofluorescence images for the flow-sorted germ cells populations. (i) Pre-meiotic cells include spermatogonia positive for CD90.2 (green). (ii) Meiosis I includes spermatocytes I at leptonema/zygonema and pachynema/diplonema stages. Synaptonemal complexes (SYCP3) are labelled in green, γH2AX in red and DNA in blue (DAPI). (iii) Meiotic II cells represent a heterogeneous population including spermatocytes II in G_1, metaphase and diakinesis. Tubulin is labelled in green and DNA in blue (DAPI). (iv) The spermiogenesis stage includes round spermatids and sperm. Legend: n, chromosome number per cell; c, number of chromatids per chromosome. Scale bar: 10 μm. (B) Highly enriched sub-populations of primary spermatocytes. (i) Flow cytometry Hoechst Blue (UV355-460/50) and Hoechst Red (UV355-670/30) plot after "in solution" immunofluorescence with DMC1 and SYCP3. Germ cells are differentiated by distinct DNA content: 4c, 2c and c. (ii) Re-gating of the 4c population displaying three different cell populations: +DMC1/+SYCP3, -DMC1/+SYCP3 and -DMC1/-SYCP3. (iii) Enrichment values for +DMC1/+SYCP3 and -DMC1/+SYCP3 sorted populations. (C) Isolation of highly enriched round spermatids and sperm through re-gating. (i) Main FACS profile from whole testis, where primary gating is established for each germline population. (ii) Isolation of round spermatids and sperm, where cells are re-gated based on how cells project a side shadow (side scatter, SSC) and a forward shadow (forward scatter, FSC), attaining highly enriched fractions of these populations. (iii) Representative images and enrichment values for round spermatids (RS) and sperm fractions after re-gating. Legend: RS – round spermatids, ES – elongated spermatids. (D) Isolation of sperm from caudate epididymis. (i) Representative cells obtained from the caudate epididymis stained with DAPI prior to FACS. (ii) Flow cytometry Hoechst Blue (UV355-460/50) and Hoechst Red (UV355-670/30) plot showing isolated sperm from caudate epididymis. Two populations of cells are evidenced (2c and c). Re-gating of the c fraction resulted in isolated sperm. (iii) Representative image and enrichment values after sorting. Legend: ES – elongated spermatids. (E) FACS profile of mouse primary fibroblast cell culture.

Figure S2

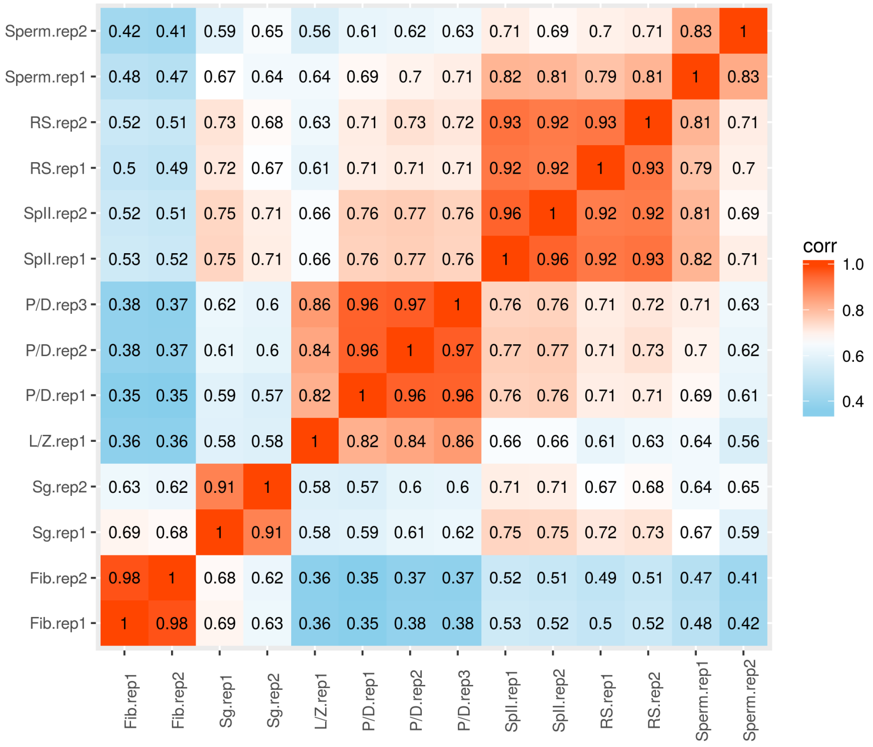

Figure S2: Correlation among Hi-C replicates, related to Figure 2. Heatmap showing the correlation values among replicates based on the pairwise similarity score calculated using HiCRep. Legend: Fib – Fibroblast, Sg – Spermatogonia, L/Z – Leptonema/Zygonema, P/D – Pachynema/Diplonema, SpII – Spermatocytes II, RS – Round Spermatids, Rep#: replicates.

Figure S3

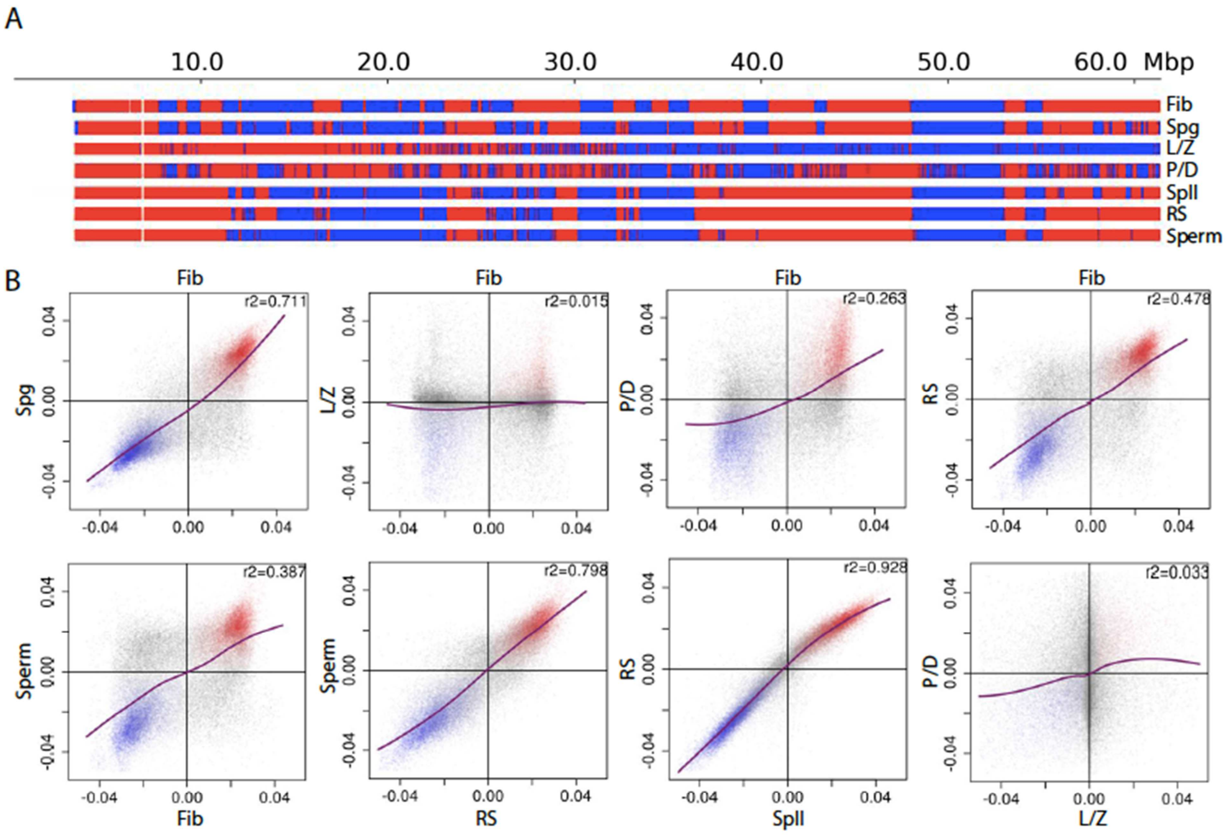

Figure S3: Compartment switching during spermatogenesis, related to Figure 3. (A) Representation of the genomic regions corresponding to A (red) and B (blue) compartments in mouse chromosome 19 for all cell types analysed. The distribution of gene density is also displayed at the bottom of the figure (purple spikes). (B) Pair-wise representation of eigenvectors between cell types genome-wide. Each dot represents a 50Kbp bin in the genome. Bins representing A compartment conservation are depicted in red, whereas in blue are depicted bins with B compartment conservation. Bins with unclear signal or compartment switching are represented in grey. The purple line is a LOESS curve showing the tendency of the compartment switching. Correlation values are represented for each pair-wise comparison. Legend: Fib – Fibroblast, Spg – Spermatogonia, L/Z – Leptonema/Zygonema, P/D – Pachynema/Diplonema, SpII – Spermatocytes II, RS – Round Spermatids.

Figure S4

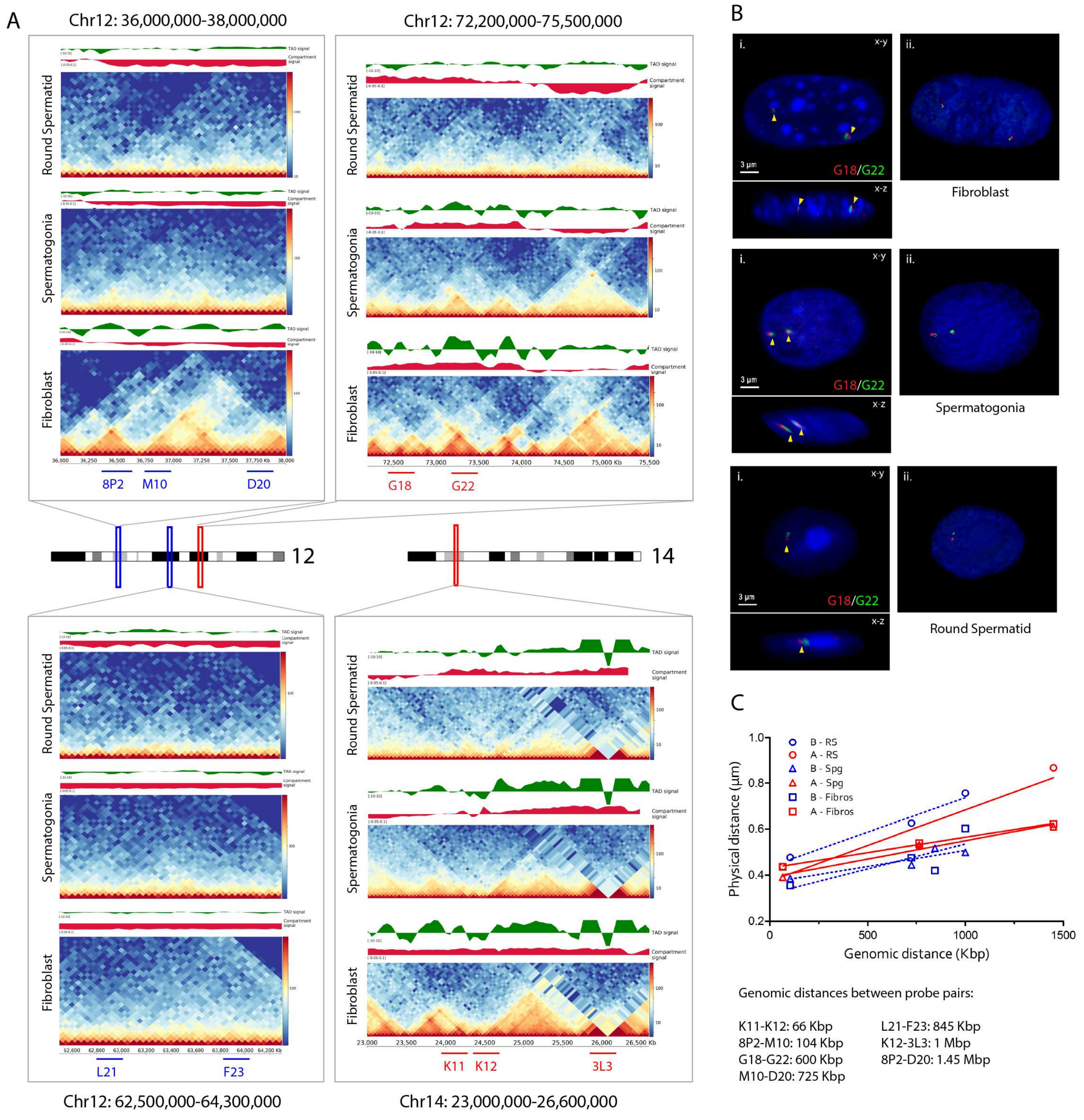

Figure S4: Analysis of A/B compartments by 3D-FISH, related to Figure 3. (A) Heatmaps from the four chromosomal regions analysed by 3D-FISH in mouse chromosomes 12 and 14. Probes located at A-compartments are red, whereas probes located at B compartments are blue. (B) Two-colour 3D-FISH images of example pairs of probes within A-compartments in fibroblasts, spermatogonia and round spermatids (see supplementary methods for probe localisation). In all cases, left panels (i) represent the confocal x-y (upper section) and x-z sections (lower section), whereas right panels (ii) represent IMARIS three-dimensional reconstructions. (C) Lineal plot of the median genomic distances (in Kbp) between pairs of probes versus physical distances (in mm) in fibroblasts (n = 275), spermatogonia (n = 155) and round spermatids (n = 97) for both types of compartments (A in red and B in blue).

Figure S5

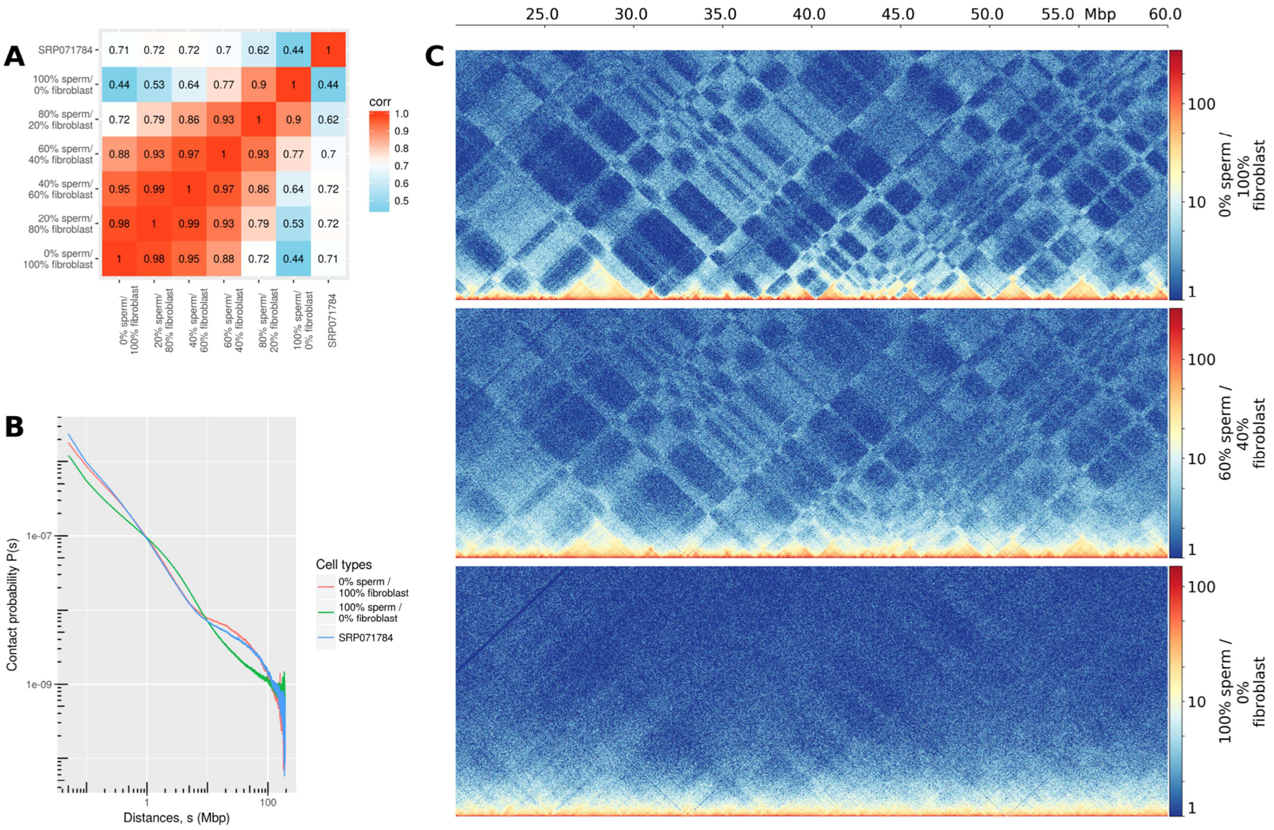

Figure S5: Simulations of samples with different fibroblast and sperm content, related to Figure 3. (A) Heatmap showing correlation values, based on the pairwise similarity score calculated using HiCRep, among samples with different fibroblast and sperm content as well as the merged sperm replicates from SRP071784 (retrieved from Jung et al. 2017). (B) Contact probability as a function of genomic distance for samples with different fibroblast and sperm content as well as the merged sperm replicates from SRP071784. (C) Chromosome 8 region-specific ICE-corrected heatmaps at 50 kbp for samples with different fibroblast and sperm content, as well as from SRP071784.

Figure S6

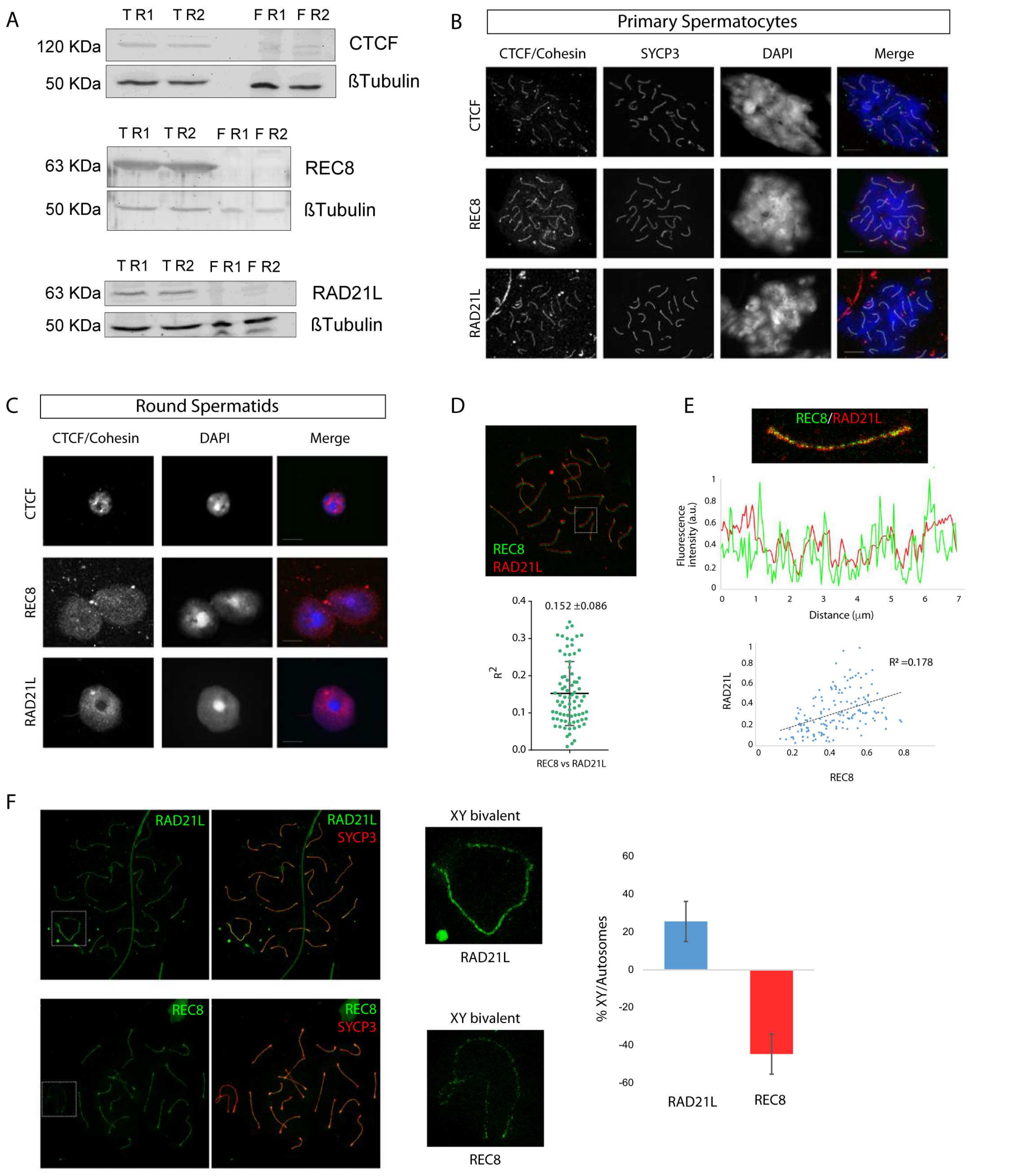

Figure S6: Expression and intracellular localisation of CTCF and cohesins (REC8 and RAD21L) in mouse germ cells, related to Figures 5 and 6. (A) Western blotting analyses on whole nuclear extracts from mouse testis (two replicates) and fibroblasts (two replicates). Abbreviations: T– testis, F –fibroblast, R# – replicate. The band corresponding to CTCF was detected at 120 KDa, REC8 and RAD21L at 63 KDa, approximately. The β-tubulin loading control was detected at about 50 kDa. (B) Examples of immunofluorescence images showing the intracellular distribution of CTCF, RAD21L and REC8 in primary spermatocytes at pachytene stage. Double immunolabelling of CTCF (red)/ RAD21L (red)/ REC8 (red) with SYCP3 (green) are shown. Scale bar: 10 μm. (C) Examples of immunofluorescence images showing the intracellular distribution of CTCF, RAD21L and REC8 in round spermatids.

Double immunolabelling of CTCF (red)/ RAD21L (red)/ REC8 (red) with SYCP3 (green) are shown. Scale bar: 10 μm. (D) Double immunolabelling of REC8 (green) and RAD21L (red) at pachytene stage showing only partial overlapping at the chromosome axes by STED microscopy. Plot of the mean correlation between REC8 and RAD21L at the autosomes (n=76 axial elements, AEs; mean ± s.d.). (E) Example of an autosome axes immunolabelled for REC8 (green) and RAD21L (red) (upper panel). Plot represents normalised signal intensity profile of REC8 (green) with RAD21L (red) (middle panel). Lower plot shows regression analysis of the correlation between REC8 and RAD21L (lower panel). (F) Example of differential immunolabelling of (i) RAD21L (increased labelling) and (ii) REC8 (decreased labelling) at the XY bivalent in a spermatocyte at pachytene. (iii) Magnification of the XY bivalent is shown for each cohesin. (iv) Plot representation of the labelling gain/loss of RAD21L and REC8 fluorescence intensity (depicted in %) along the XY axes in comparison with autosomes (n=19 cells).

Figure S7

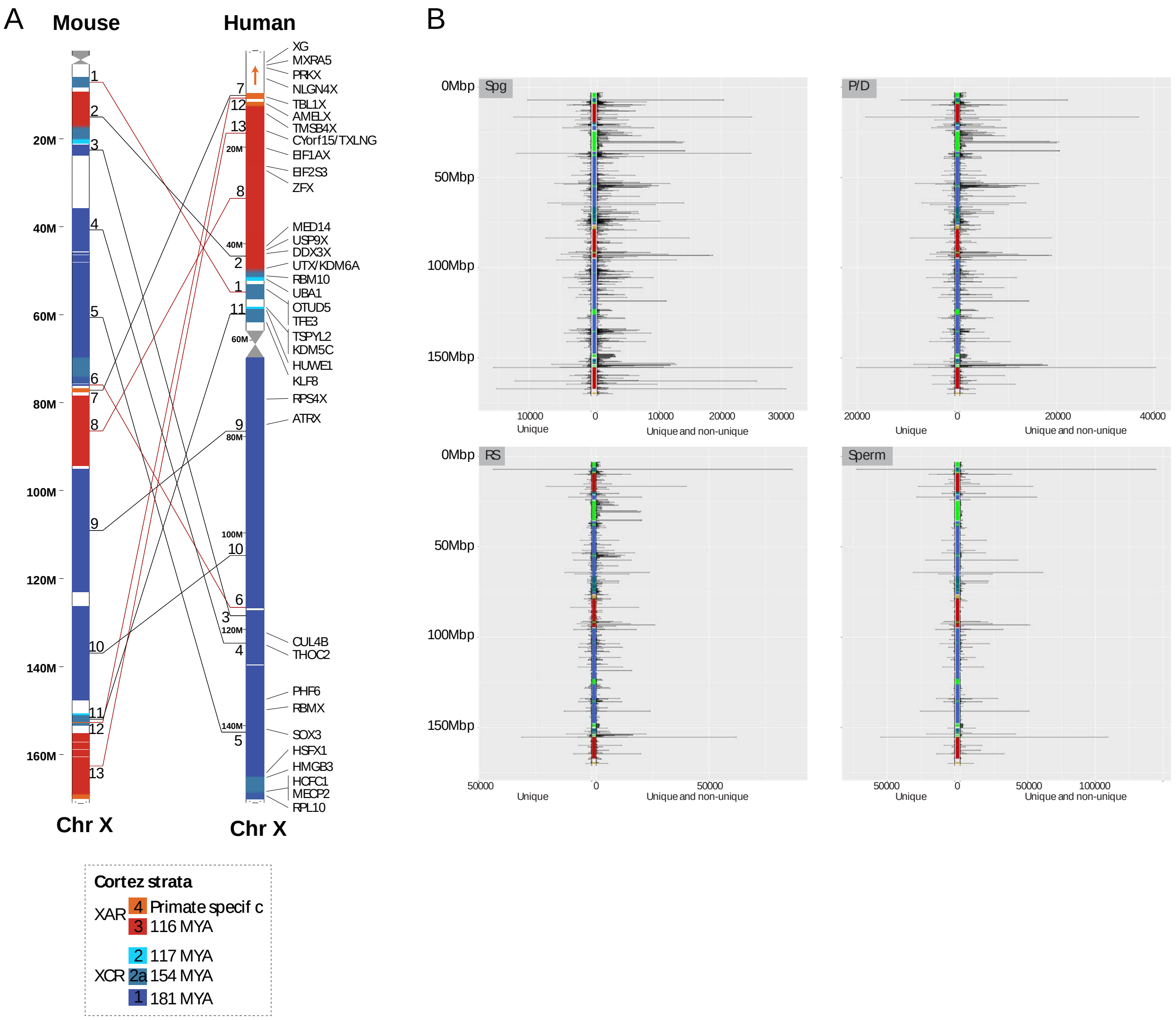

Figure S7: Evolutionary strata and ampliconic regions in the mouse X chromosome, related to Figure 7. (A) Evolutionary strata (from Cortez et al., 2014) for the human and mouse X chromosomes. Strata were assigned to the mouse X using human synteny (https://ensembl.org/Homo_sapiens/Location/Synteny?r=X). White regions are breaks in synteny that usually correspond to amplicons (see panel B). (B) RNA-seq tag counts on the X chromosome in spermatogonia (Spg), pachynema/diplonema (P/D), round spermatids (RS) and sperm. Colours in the centre represent evolutionary strata (as in panel A), green represent amplicons (see Table S7 for coordinates). Peaks to the left represent counts for uniquely mapped RNA-seq reads. Peaks to the right are counts for both uniquely mapped and multi-mapped RNA-seq reads. As expected, in the ampliconic regions counts are higher when including multi-mapped reads.
