# Supplementary material for: Three-Dimensional Genomic Structure and Cohesin Occupancy Correlate with Transcriptional Activity during Spermatogenesis
Source: Cell Rep. 2019 Jul 9;28(2):352–367.e9. doi: 10.1016/j.celrep.2019.06.037 (PMC6635386; doi:10.1016/j.celrep.2019.06.037)
Supplement: Document S2. Article plus Supplemental Information [file mmc10.pdf]

Cell Reports

Article

Three-Dimensional Genomic Structure and Cohesin Occupancy Correlate with Transcriptional Activity during Spermatogenesis

Graphical Abstract

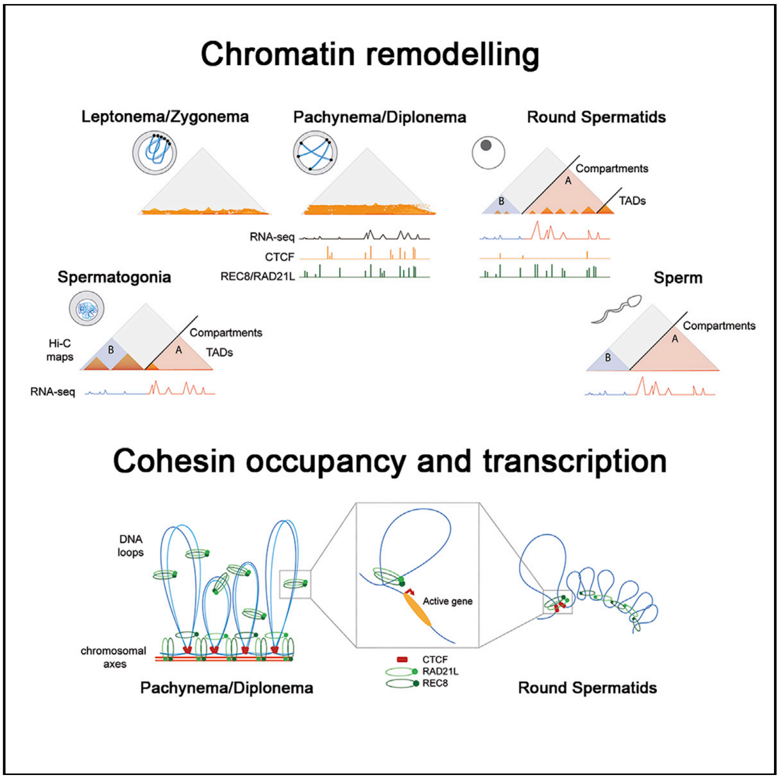

Highlights

- Meiotic and post-meiotic cells differ in their hierarchal genome organization
- Meiotic cohesin loading correlates with gene expression and local insulation
- Sperm cells contain compartments with active transcription but no clear TADs
- Genes that escape X chromosome silencing associate with promoter cohesin occupancy

Authors

Covadonga Vara, Andreu Paytuví-Gallart, Yasmina Cuartero, ..., Paul D. Waters, Marc A. Marti-Renom, Aurora Ruiz-Herrera

Correspondence

martirenom@cnag.crg.eu (M.A.M.-R.), aurora.ruizherrera@uab.cat (A.R.-H.)

In Brief

The formation of mammalian germ cells involves dramatic chromosomal movements and chromatin remodeling, whose regulatory pathways are far from understood. Vara et al. show how the dynamics of insulator proteins' occupancy and transcriptional activity are coupled during the 3D genome re-organization that takes place during mouse spermatogenesis.

Vara et al., 2019, Cell Reports *28*, 352–367
July 9, 2019 © 2019 The Author(s).
https://doi.org/10.1016/j.celrep.2019.06.037

CellPress

OPEN ACCESS
CellPress

Cell Reports
Article

Three-Dimensional Genomic Structure and Cohesin Occupancy Correlate with Transcriptional Activity during Spermatogenesis

Covadonga Vara,[1,2,12] Andreu Paytuví-Gallart,[1,2,3,12] Yasmina Cuartero,[4,5,12] François Le Dily,[4,5] Francisca Garcia,[6] Judit Salvà-Castro,[1,2] Laura Gómez-H,[7] Eva Julià,[4,8] Catia Moutinho,[5] Riccardo Aiese Cigliano,[3] Walter Sanseverino,[3] Oscar Fornas,[4,9] Alberto M. Pendás,[7] Holger Heyn,[5,9] Paul D. Waters,[10] Marc A. Marti-Renom,[4,5,9,11,*] and Aurora Ruiz-Herrera[1,2,13,*]

[1]Departament de Biologia Cel.lular, Fisiologia i Immunologia, Universitat Autònoma de Barcelona (UAB), Cerdanyola del Vallès 08193, Spain
[2]Genome Integrity and Instability Group, Institut de Biotecnologia i Biomedicina (IBB), Universitat Autònoma de Barcelona (UAB), Cerdanyola del Vallès 08193, Spain
[3]Sequentia Biotech, Carrer Comte D'Urgell 240, Barcelona 08036, Spain
[4]Centre for Genomic Regulation (CRG), The Barcelona Institute for Science and Technology (BIST), Carrer del Doctor Aiguader 88, Barcelona 08003, Spain
[5]CNAG-CRG, Centre for Genomic Regulation, The Barcelona Institute of Science and Technology (BIST), Baldiri Reixac 4, Barcelona 08028, Spain
[6]Unitat de Cultius Cel.lulars, Universitat Autònoma de Barcelona (UAB), Cerdanyola del Vallès 08193, Spain
[7]Molecular Mechanisms Program, Centro de Investigación del Cáncer and Instituto de Biología Molecular y Celular del Cáncer (CSIC-Universidad de Salamanca), Salamanca 37007, Spain
[8]Institut Hospital del Mar d'Investigacions Mèdiques (IMIM), Carrer del Doctor Aiguader 88, PRBB Building, Barcelona 08003, Spain
[9]Pompeu Fabra University (UPF), Doctor Aiguader 88, Barcelona 08003, Spain
[10]School of Biotechnology and Biomolecular Sciences, Faculty of Science, UNSW Sydney, NSW 2052, Australia
[11]ICREA, Pg. Lluís Companys 23, Barcelona 08010, Spain
[12]These authors contributed equally
[13]Lead Contact
*Correspondence: martirenom@cnag.crg.eu (M.A.M.-R.), aurora.ruizherrera@uab.cat (A.R.-H.)
https://doi.org/10.1016/j.celrep.2019.06.037

SUMMARY

Mammalian gametogenesis involves dramatic and tightly regulated chromatin remodeling, whose regulatory pathways remain largely unexplored. Here, we generate a comprehensive high-resolution structural and functional atlas of mouse spermatogenesis by combining *in situ* chromosome conformation capture sequencing (Hi-C), RNA sequencing (RNA-seq), and chromatin immunoprecipitation sequencing (ChIP-seq) of CCCTC-binding factor (CTCF) and meiotic cohesins, coupled with confocal and super-resolution microscopy. Spermatogonia presents well-defined compartment patterns and topological domains. However, chromosome occupancy and compartmentalization are highly re-arranged during prophase I, with cohesins bound to active promoters in DNA loops out of the chromosomal axes. Compartment patterns re-emerge in round spermatids, where cohesin occupancy correlates with transcriptional activity of key developmental genes. The compact sperm genome contains compartments with actively transcribed genes but no fine-scale topological domains, concomitant with the presence of protamines. Overall, we demonstrate how genome-wide cohesin occupancy and transcriptional activity is associated with three-dimensional (3D) remodeling during spermatogenesis, ultimately reprogramming the genome for the next generation.

INTRODUCTION

Mammalian genomes are packaged into a tailored chromatin structure, the regulation of which depends on several superimposed layers of organization, including epigenetic modifications (of both the DNA and nucleosomes) and the higher-order organization of chromatin compartments inside the nucleus. This organization is achieved by chromatins folding into loops, topologically associating domains (TADs), and compartments (A and B), which can ultimately influence transcriptional activity (Dixon et al., 2012; Lieberman-Aiden et al., 2009; Rao et al., 2014). How these different levels of chromatin organization interact during the cell cycle has just begun to be elucidated (Dekker et al., 2013). In somatic cells, the highly compartmentalized folding of the genome in interphase is lost during mitosis, when chromosomes are linearly organized in consecutive chromatin loops (Gibcus et al., 2018; Naumova et al., 2013). Recent studies in mice have suggested remarkable chromatin architecture reprogramming during the formation of germ cells (Alavattam et al., 2019; Patel et al., 2019; Wang et al., 2019) and early development (Du et al., 2017; Flyamer et al., 2017). However, how the higher-order chromatin organization is configured during all stages of spermatogenesis, and how insulator proteins and

 © 2019 The Author(s).
This is an open access article under the CC BY license (http://creativecommons.org/licenses/by/4.0/).

cohesins determine this organization to regulate transcription activity, remains largely unexplored.

Germ cells represent a unique cell model, where unipotent diploid cells (gonia) undergo extensive cellular differentiation (gametogenesis) to form highly differentiated cells (oocytes and sperm) that ultimately form a totipotent embryo. In the case of mammalian males, this complex process is divided into three stages: (1) proliferation and differentiation of spermatogonia (Spg); (2) meiosis, a reductional division that produces haploid cells through two consecutive cell divisions (meiosis I and meiosis II); and (3) spermiogenesis, where round spermatids (RSs) are transformed into densely compacted spermatozoa. These sequential developmental stages involve dramatic and tightly regulated chromosomal re-organization and chromatin remodeling. It is during the first meiotic prophase (prophase I) that homologous chromosomes align, pair, synapse, and recombine. All these processes are interconnected through four sequential stages: leptonema, zygonema, pachynema, and diplonema (Handel and Schimenti, 2010).

At leptonema, chromosomes cluster by their telomeres to the nuclear envelope in the bouquet (Reig-Viader et al., 2016). This structure promotes the pairing of homologous chromosomes by the formation of proteinaceous structures along chromosomes formed by cohesins (i.e., REC8 and RAD21L; Gutiérrez-Caballero et al., 2011; Llano et al., 2012) and proteins of the synaptonemal complex (SC). Meiotic recombination is triggered by the formation of double strand breaks (DSBs), caused by the endonuclease protein SPO11 (Keeney et al., 1997). DSBs are then initiated at zygonema, leading to synapsis between homologous chromosomes. Subsequently, at pachynema, SCs are completely established, creating bivalent structures with resolved recombination producing crossover events. At diplonema, homologous chromosomes start to segregate by the disassembly of SCs to produce spermatocytes II, which undergo a second meiotic division resulting in RSs. Finally, spermatids become sperm ready for fecundation through spermiogenesis, a differentiation stage that includes changes in cell morphology and DNA packaging via the replacement of histones by protamines (testis-specific histone variants).

Despite recent analysis of genome conformational changes in male germ cells (Alavattam et al., 2019; Patel et al., 2019; Wang et al., 2019), how the higher hierarchal level of genome organization is related to gene expression and insulator proteins during spermatogenesis remains unknown. It has been generally accepted that there are two waves of active transcription: one before entering meiosis and a second in the transition from RSs to sperm (Sassone-Corsi, 2002). However, recent transcriptome analysis in germ cells suggests that these two transcriptional waves might take place earlier (da Cruz et al., 2016), which evidences a finely tuned regulation of chromatin remodeling and active transcription. Here, we implement a reproducible flow cytometry protocol to isolate enriched male mouse germ cell populations representing all stages of spermatogenesis: premeiotic (Spg), meiotic (leptonema, zygonema, pachynema, and diplonema), and post-meiotic cells (RSs and sperm). On these sorted germ cell populations, we performed genome-wide chromosome conformation capture analysis (*in situ* Hi-C [chromosome conformation capture sequencing]), coupled with RNA sequencing (RNA-seq) and chromatin immunoprecipitation sequencing (ChIP-seq) of CCCTC-binding factor (CTCF) and meiotic cohesins (Figure 1A). These data have permitted the comprehensive study of the close interplay between chromatin higher-order organization dynamics and function during mouse spermatogenesis.

RESULTS

Dynamic Overall Chromatin Structure Reorganization during Spermatogenesis

To unveil changes in chromosome conformation during spermatogenesis, we developed a reproducible fluorescence-activated cell sorting (FACS) protocol to obtain, based on DNA content and chromatin complexity, highly enriched (90.4% average enrichment) cell fractions for Spg, primary spermatocytes at the leptonema-zygonema (L/Z) and pachynema-diplonema (P/D) stages, RSs, and sperm (Figures 1B, 1C, and S1) (STAR Methods). For each germ cell fraction, as well as for a mouse primary fibroblast cell line as a somatic profile, we performed *in situ* Hi-C (Rao et al., 2014) (Figures 1D–1F and S1). After filtering the raw Hi-C interactions, an average of 237.86 million valid interactions were obtained per cell type (Tables S1 and S2). The comparison between biological replicates resulted in highly reproducible Hi-C maps (Figure S2).

Genome organization changed during spermatogenesis (Figures 1F and 2A–2F), as reflected by the analysis of distance-dependent interaction frequencies (Figure 2C) and inter- and intra-chromosomal interaction ratios (Figure 2D). Fibroblasts showed high inter- and intra-chromosomal interaction ratios (>0.6) inversely correlated with chromosomal size ($p < 0.001$), suggesting distinct chromosomal compartmentalization within nuclei (Figure 2D). In contrast, inter- and intra-chromosomal interaction ratios decreased 2-fold to about 0.3 for all chromosomes in Spg, suggesting that a commitment to enter meiosis is accompanied by a drastic remodeling of chromosomal territories within the nucleus. Importantly, this decrease in inter- and intra-chromosomal interactions was concomitant with the dynamic changes of the so-called A-B compartments (Lieberman-Aiden et al., 2009). Likewise, fibroblasts and Spg, both in interphase, shared similar contact probability patterns at short distances (from 0.5 to 7 Mbp) and had the lowest intermediate interactions (between 1 and 10 Mb) (Figure 2C). However, at larger genomic distances (>10 Mb), fibroblasts showed a slight change in the slope at 10 Mbp with fewer interactions, whereas Spg maintained the same trend up to 100 Mbp.

As meiosis progressed, compartments were mostly lost in primary spermatocytes (L/Z and P/D), coinciding with prophase I, when homologous chromosomes condensate, align, pair, synapse, and recombine (Figures 2B, 3A, and 3B). Consistent with this absence of compartments during prophase I, eigenvector values were close to 0 (Figures 2B, 3A, and 3B), and inter- and intra-chromosomal interaction ratios reached a minimum for all chromosomes (Figure 2D). An exception was the sex chromosomes in P/D, which had detectable variations in the inter- and intra-chromosomal interactions ratio. This unusual pattern most likely reflects meiotic sex chromosome inactivation (MSCI), the process by which the X and Y chromosomes

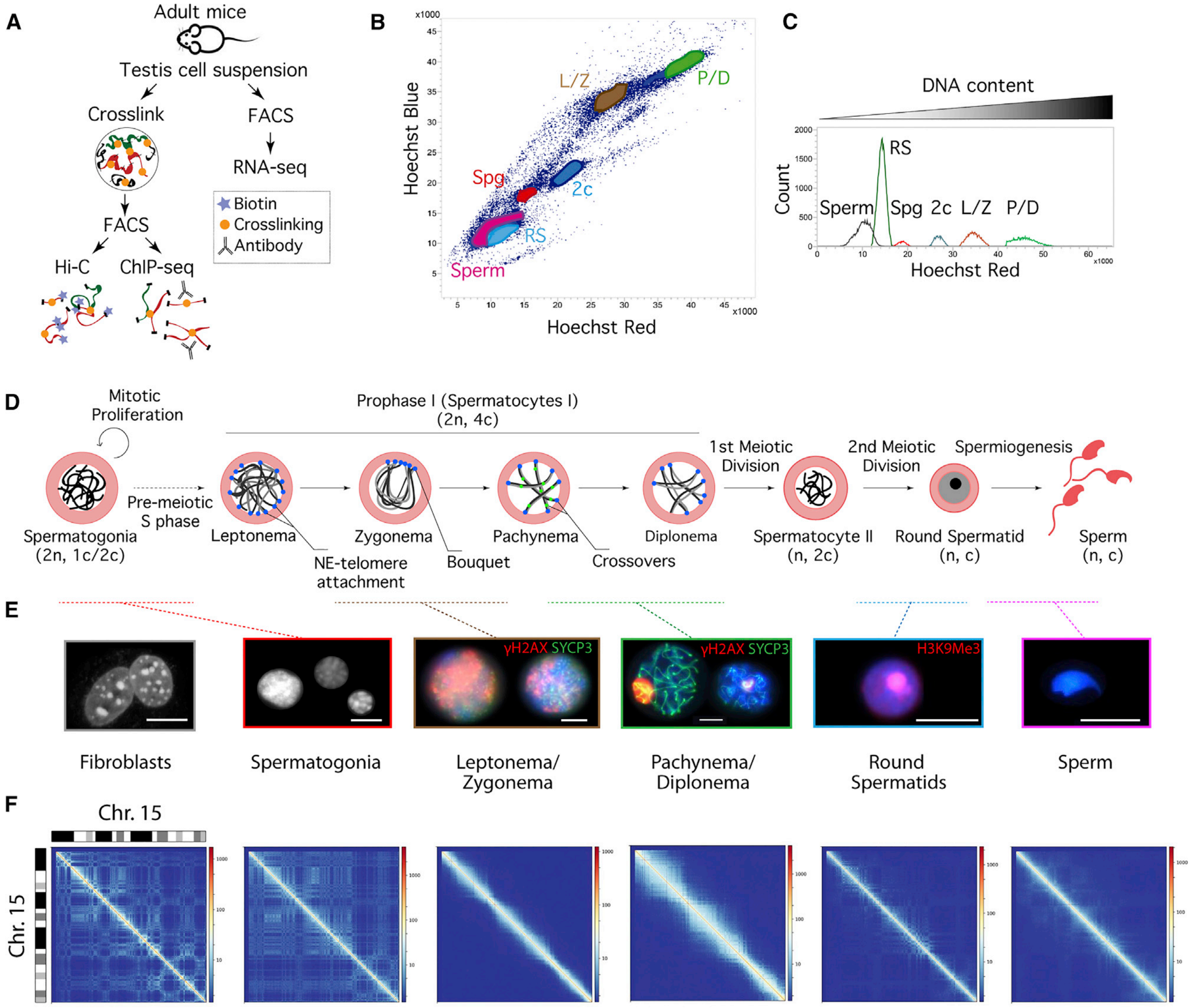

Figure 1. Higher-Order Chromatin Structure during Spermatogenesis

(A) Experimental workflow: Hi-C, ChIP-seq, and RNA-seq on fluorescence-activated cell sorting (FACS)-enriched mouse germ cells.

(B) Flow cytometry Hoechst Blue (UV355-460/50) and Hoechst Red (UV355-670/30) plot showing spermatogonia (Spg), leptonema-zygonema (L/Z), pachynema-diplonema (P/D), secondary spermatocytes (2c), round spermatids (RSs), and sperm. Recovered germ cell fractions presented the following average enrichment: 91% for Spg, 88.7% for L/Z, 89.2% for P/D, 92.9% for RS, and 90% for sperm.

(C) Histogram of the differential DNA content showing cell events for each FACS-isolated germ cell population (Hoechst Red, UV355-670/30).

(D) Overview of the spermatogenesis process (adapted from Reig-Viader et al., 2016). Numbers between parentheses indicate the diploid (2n) haploid (n) number for each cell type and the number of chromatids per chromosome (4c, 2c, or c).

(E) Representative immunofluorescence images showing DAPI-stained DNA (gray/blue) and specific meiotic proteins for the different cell populations included in the present study. Fibroblasts and Spg have DAPI-stained DNA in gray. For L/Z and P/D, DAPI is shown in blue, SYCP3 in green, and γH2AX in red. The image represents a mosaic of two individual captured cells. In RSs, DAPI is blue, and H3K9me3 (marker for the constitutive heterochromatin at centromeres- chromocenters) is red. Scale bars, 10 um.

(F) Iterative correction and eigenvector decomposition (ICE)-corrected Hi-C matrices for chromosome 15 at a 50-kbp resolution for the cell types analyzed. Deep blue lines indicate non-mapped bins.

See also Figure S1.

are transcriptionally inactivated in primary spermatocytes, forming the sex body at the periphery of the nucleus (Turner, 2007). Another structural feature characteristic of early meiosis that was revealed in our analyses was the bouquet structure. We detected high inter-chromosomal contact between telomeres in primary spermatocytes, being more prominent in L/Z (Figures 2E and 2F). Both L/Z and P/D had an enrichment of counts at the most proximal sub-telomeric bins, but cells at L/Z had significantly higher interactions (Wilcoxon test, $p < 0.05$) than any other cell type (Figures 2E and 2F). The analysis of the

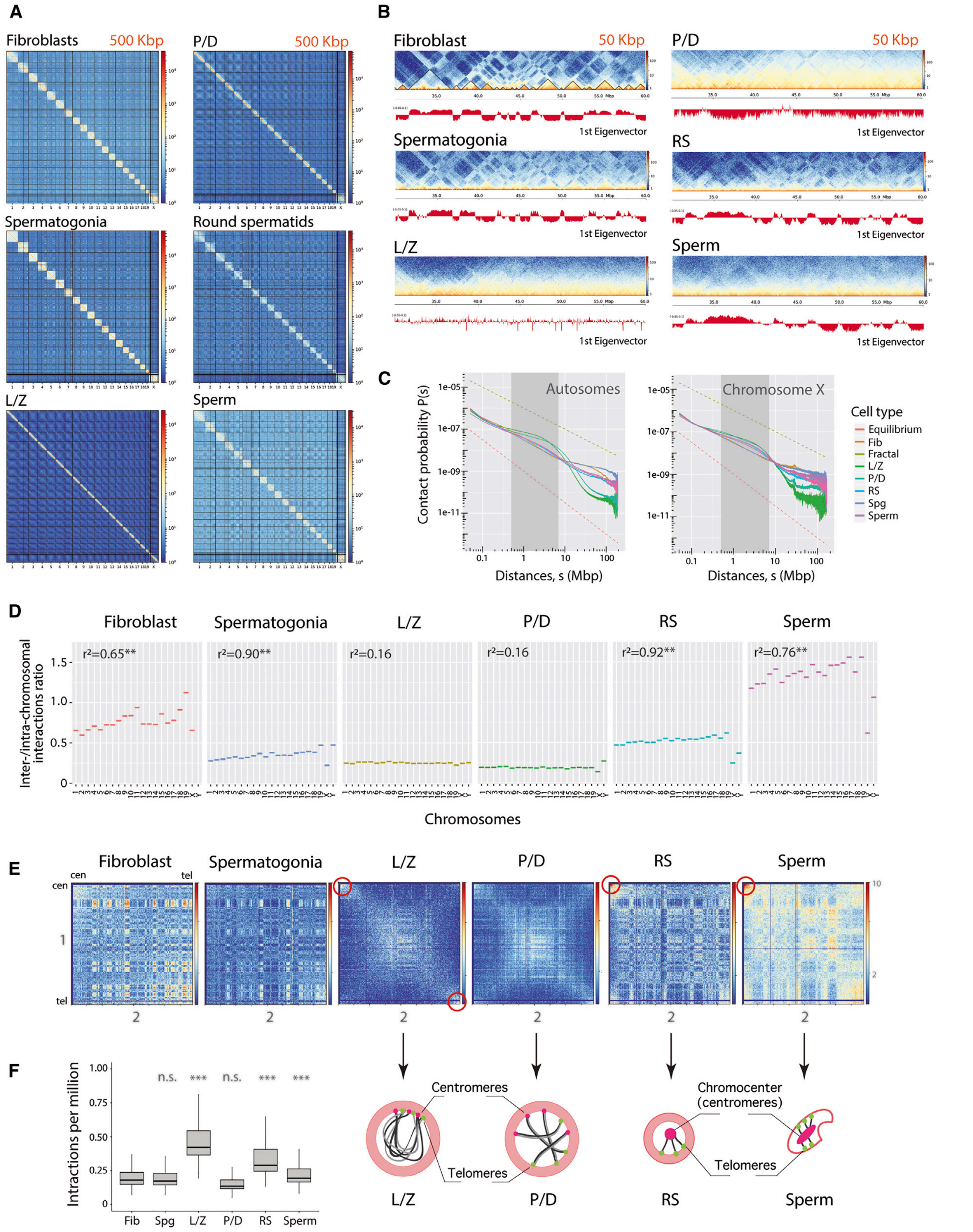

(legend on next page)

distance-dependent interaction frequencies revealed a distinct chromosome organization for primary spermatocytes, with two abrupt changes in slope: the first at 2.5−4.5 Mbp and the second at 40 Mbp (Figure 2C).

After meiosis, haploid cells (RSs and sperm) had a distinctive higher-order chromatin structure. Although A-B compartments re-appeared after being lost in previous stages, they presented as a blurry plaid pattern of larger mean size (0.86 Mbp in RSs and 0.93 Mbp in sperm) compared to Spg (Figures 2F, 3B, S3A, and S3B; Table S3). Interestingly, the proportion of genomic bins with the same compartment status (A or B) was higher between haploid cells (r^2 = 0.80) than between haploid cells and Spg (RSs versus Spg, r^2 = 0.58; sperm vs Spg, r^2 = 0.48). This pattern of differential compartmentalization in RSs was also validated by three-dimensional fluorescence *in situ* hybridization (3D-FISH), where physical distances between pairs of loci increased compared to Spg and fibroblasts (Figure S4; Table S4). Moreover, the inter- and intra-chromosomal interaction ratio values were higher in RSs for all chromosomes, with the exception of sex chromosomes (Figure 2D), which are known to form post-meiotic sex chromatin (PMSC) attached to the chromocenters (Namekawa et al., 2006). Thus, the formation of PMSC correlates with chromatin remodeling that results in low inter- and intra-chromosomal interactions. Remarkably, in sperm, inter-chromosomal interactions were greater than intra-chromosomal interactions, compared to previous stages, and inversely correlated with chromosomal size ($p < 0.001$) (Figure 2D). Since ratio values were higher than in fibroblasts, the higher-order chromatin structure is likely densely packed in sperm but remains in chromosome territories. The sex chromosomes did not follow the autosomal pattern; the interactions ratio decreased by 2-fold (Figure 2D). In both haploid cell types, we detected higher inter-chromosomal contact between centromeres, suggestive of the presence of the chromocenters (Figures 2E and 2F). In fact, it is known that in mouse sperm, centromeres are located at the center of the nucleus, while telomeres attach to the nuclear envelope (Haaf and Ward, 1995). This particular compartmentalization in both cell types was also reflected by a decrease in interaction frequencies as genomic distance increased (Figure 2C).

These observations indicate that the genome suffers a major structural re-organization during spermatogenesis with dynamic and dramatic changes in chromosome occupancy and compartments.

Two Rounds of TAD Reorganization in Primary Spermatocytes and Sperm

To further investigate the dynamics of the higher-order chromatin structure at the sub-megabase scale, we identified TADs and examined the robustness of their boundaries using TADbit (Serra et al., 2017) at 50-Kbp resolution. Similar to the A-B compartment patterns, TADs were well defined in both fibroblasts and Spg (Figure 3C). In primary spermatocytes, however, there was a substantial reduction in the variance of TAD insulation score as well as an increase in TAD size, especially in L/Z (Figures 3C−3E). TAD insulation scores were partially recovered in RSs but, in contrast to previous observations (Jung et al., 2017; Wang et al., 2019), were drastically reduced in sperm cells (close to 0) (Figures 3D, 3E, and S5).

A total of 2002 TADs, with an average length of 1.3 Mbp, were identified in fibroblasts, which was more than in Spg (834 TADs, mean size of 3.26 Mbp) (Figure 3C; Table S3). Although fewer TADs were identified in primary spermatocytes (305 TADs in L/Z and 294 TADs in P/D), their boundaries had high strength scores (74.25% of TADs in L/Z, and 79.59% in P/D, had scores >9; Table S3). This pattern contrasted with RSs, which had a large number of small TADs (n = 4,649) with low border strength scores (Figure 3C; Table S3). Meta-border plots confirmed this dynamic (Figure 3F). These results demonstrate that TADs also underwent a large reorganization in genome structure during spermatogenesis, concomitant to larger changes in chromosome territories and compartments.

In summary, our data suggest at least four distinct patterns of chromatin interactions during spermatogenesis progression: (1) interphase-like organization (e.g., Spg); (2) a condensed pattern in prophase I (i.e., L/Z and P/D) where A-B compartments and TADs are largely lost; (3) RSs with a blurry compartment plaid pattern; and (4) sperm where TADs are reduced, but A-B compartments are observed.

Functional Compartment Switching during Spermatogenesis

To assess whether A-B compartmentalization changes during spermatogenesis correlated with differential expression of resident genes, we investigated changes of compartment type between the stages where compartments could be clearly observed. The proportion of the genome organized in the

Figure 2. Chromosomal Organization in Interphase, Pre-meiotic, Meiotic, and Post-meiotic Cells

(A) Genome-wide ICE-corrected heatmaps at 500 kbp for the cell types analyzed.

(B) Chromosome 18 region-specific ICE-corrected heatmaps at 50 kbp (from 20 Mbp to 60 Mbp), depicting compartment signal (first eigenvector) for all cell types.

(C) Contact probability P(s) as a function of genomic distance in all cell types for autosomes (left panel) and the X chromosome (right panel). Discontinuous straight lines correspond to the fractal (green) and equilibrium (red) models (Lieberman-Aiden et al., 2009). Gray-shadowed area expands the genomic region from 0.5 to 7 Mbp.

(D) Inter- and intra-chromosomal interaction ratios for each chromosome and cell type. Correlation values (**$p < 0.001$) between the inter- and intra-chromosomal ratio and chromosomal size (autosomes only) are shown for each cell type.

(E) Heatmaps showing normalized inter-chromosomal interactions between chromosomes 1 and 2 in all cell types. Red circles represent high-contact regions.

(F) Left panel: boxplots depicting inter-chromosomal interactions per million at sub-centromeric regions (from the centromere up to 3.5 Mbp) for all cell types (Wilcoxon test, **** < 0.0001; n.s., not statistically significant when compared to fibroblasts). Right panel: schematic representation of chromosomes and centromeres and telomeres in L/Z, P/D, RSs, and sperm. Dots represent centromeres (pink) and telomeres (green). In RSs and sperm, all centromeres associate in the center of the cell forming the chromocenter.

Fib, fibroblast; Spg, spermatogonia; L/Z, leptonema-zygonema; P/D, pachynema-diplonema; RS, round spermatids; cen, centromeres of acrocentric chromosomes; tel, telomeres of non-centromeric ends. See also Figures S2–S5.

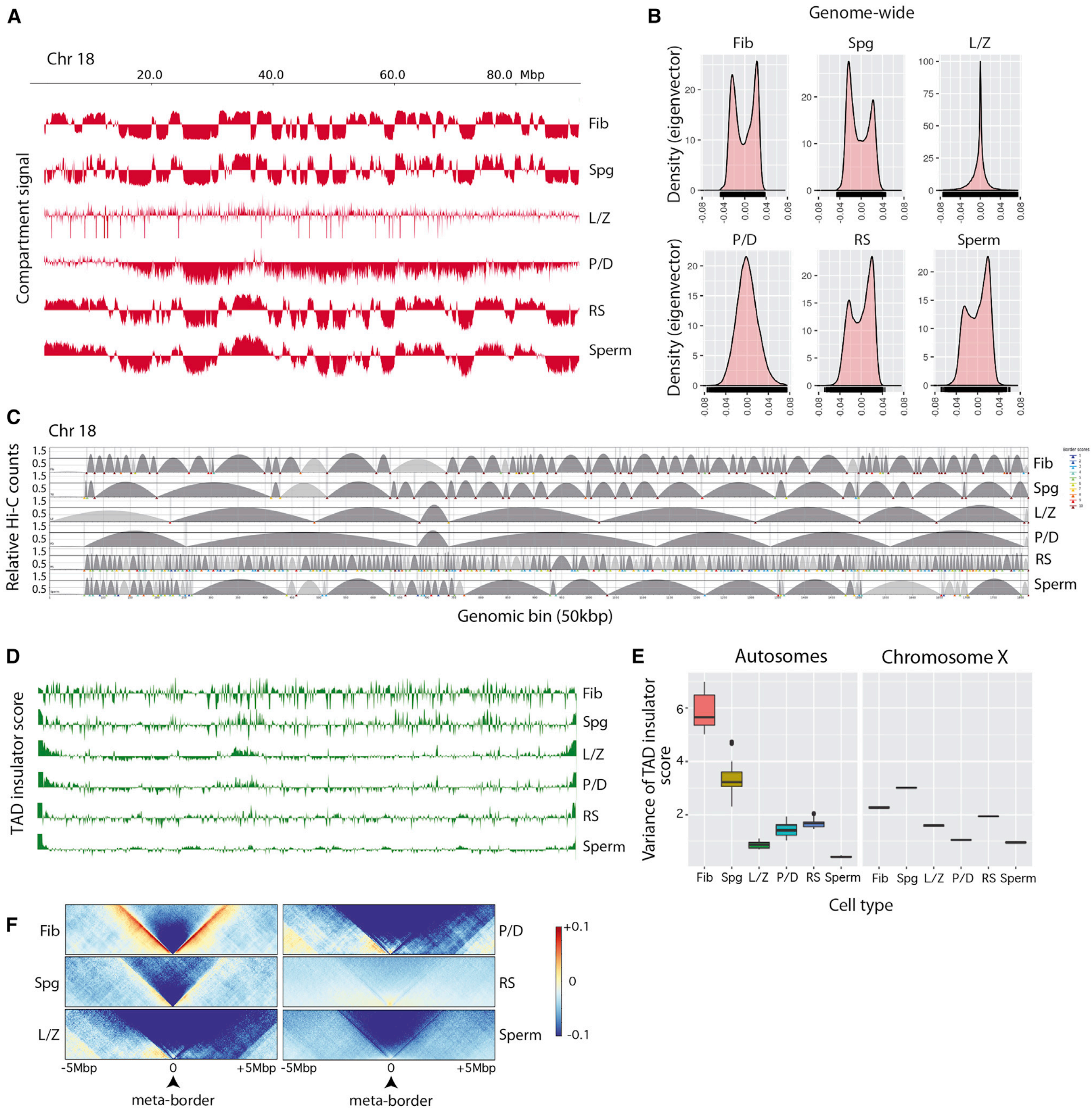

Figure 3. Chromosome-specific A-B Compartment Profiles and TAD Signals

(A) Compartment signal (first eigenvector) across chromosome 18.

(B) Density plots of eigenvector values considering autosomes.

(C) TAD border alignments along chromosome 18. Dark gray arches represent TADs with higher intra-TAD interactions than expected. TAD border robustness (from 1 to 10) is represented by a color gradient.

(D) Representation of TAD insulator score in mouse chromosome 18.

(E) Variance of the TAD insulation scores for autosomes (left panel) and the X chromosome (right panel).

(F) Meta-plots for all TAD boundaries detected in fibroblasts (n = 2002), Spg (n = 834), L/Z (n = 305), P/D (n = 294), RSs (n = 4649), and sperm (n = 1042).

Fib, fibroblast; Spg, spermatogonia; L/Z, leptonema-zygonema; P/D, pachynema-diplonema; SpII, spermatocytes II; RS, round spermatids. See also Figures S3–S5.

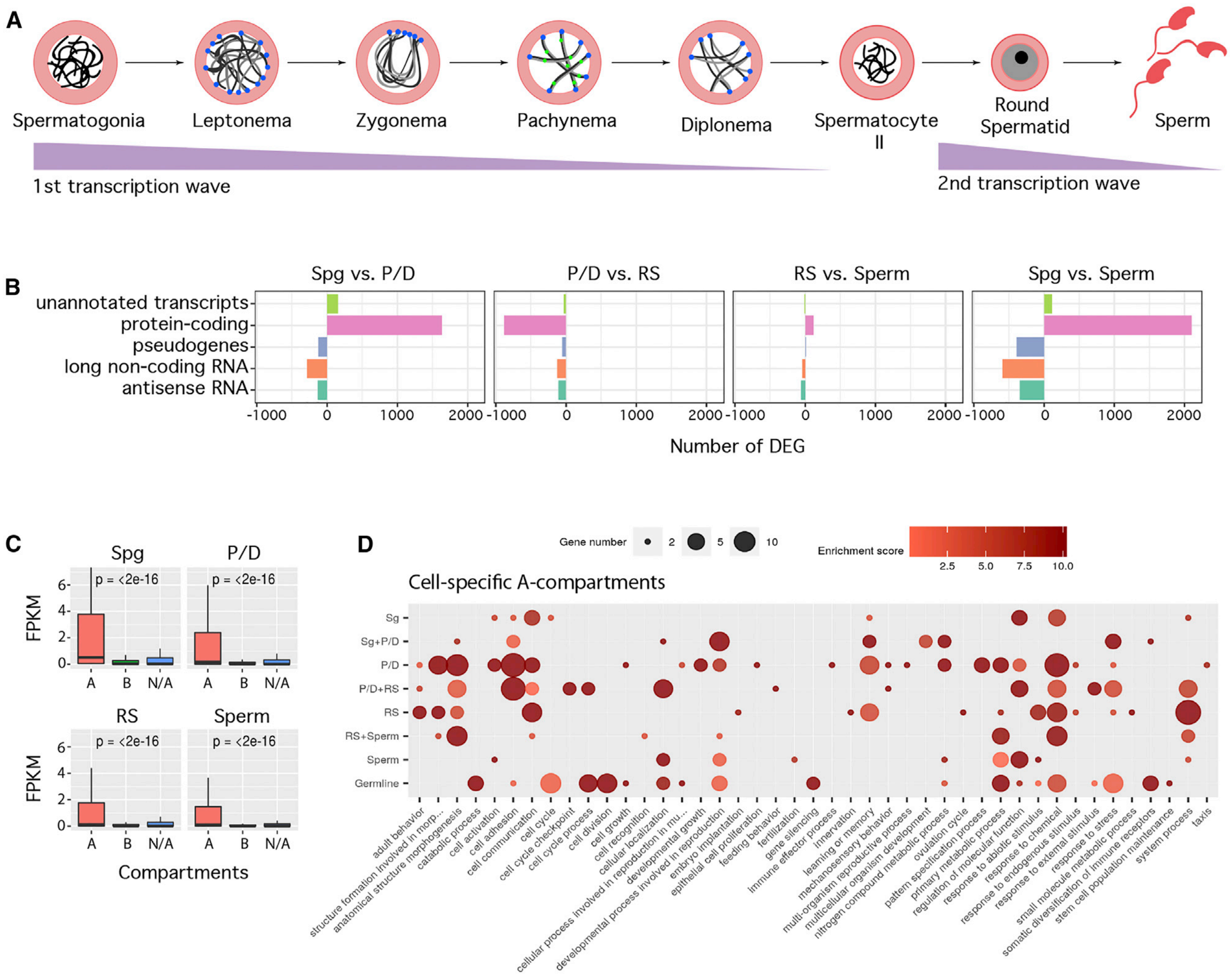

Figure 4. Dynamics of Gene Expression during Gametogenesis

(A) Two extensive waves of transcription take place during spermatogenesis.

(B) Differentially expressed genes (DEGs) for each pairwise comparison, including known nuclear protein-coding RNA (RefSeq), long noncoding RNA, antisense RNA, processed transcripts, and mitochondrial nuclear protein-coding RNA.

(C) Boxplots representing genome-wide expression (as fragments per kilobase of transcript per million mapped reads [FPKM] values) according to A-B compartment assignment (N/A, not assigned compartments).

(D) Gene Ontology (GO) analysis of expressed genes in cell-specific A compartments. Bubble size represents the number of genes assigned to each GO. Only GO terms with two or more genes are represented.

Spg, spermatogonia; P/D, pachynema-diplonema; RS, round spermatids.

A compartment was 45.7% in fibroblast and reduced to 39.4% in Spg, before a rise in RSs (46.9%) and sperm (48.6%). Since A compartments correlate with open chromatin and active genes (Lieberman-Aiden et al., 2009), the switching of compartment types can provide insight into genome function during spermatogenesis. We therefore performed low-input RNA-seq (four biological replicates) on a selected group of the enriched germ cell populations: Spg, primary spermatocytes (P/D), RSs, and sperm. After filtering, an average of 35.37 million paired-end reads was obtained per cell type (Table S5).

The total number of expressed genes decreased as spermatogenesis progressed, with 19,145 expressed in Spg, 15,480 in P/D, 14,706 in RSs, and 13,646 in sperm. Pairwise differential gene expression analysis between cell types generated lists of differentially expressed genes (DEGs), which were classified as (1) protein-coding RNA; (2) long non-coding RNA (lncRNA); (3) antisense RNA (asRNA); (4) processed and unprocessed pseudogenes; or (5) unannotated and unconfirmed transcripts (Figure 4; Table S6). The majority (76.55%) of DEGs were protein-coding, with their abundance reducing as spermatogenesis progressed. This was coupled with an increased expression of lncRNAs and non-coding asRNAs and pseudogenes (Figure 4; Table S6). Consistent with the global shutdown in gene expression that occurs during meiosis (Sassone-Corsi, 2002), the net balance between pairwise comparisons (Spg versus P/D, RSs versus sperm, and Spg versus sperm) was negative, which

suggests higher transcriptional activity in RSs than in primary spermatocytes (Figures 4A and 4B).

Consistent with a correlation between chromatin remodeling and active transcription, genes in the A compartments had significantly higher expression than those in the B compartments (Figures 4C and 4D). As expected, genes related to spermatogenesis (e.g., morphogenesis and cell differentiation) were enriched in the A compartments (Figure 4D). In RSs, during the second wave of transcription (Figure 4A), the most representative Gene Ontology (GO) term was "system process," which included 27 genes involved in sensory perception, including olfactory receptors. Likewise, genes with important roles in fertilization were also expressed in RSs, including the acrosome reaction (e.g., *Plcz1* and *Smcp*). Therefore, the transformation of RSs into spermatozoa was accompanied by the transcription of genes related to spermiogenesis and sperm function located in newly created A compartments.

Differential CTCF and Cohesin Loading Correlates with Gene Expression and Chromatin Remodeling

Western blots confirmed the presence of CTCF and meiotic cohesins (REC8 and RAD21L) in whole-testis protein extracts. Immunofluorescence (IF) then revealed a previously unreported pattern for CTCF, with signal along all chromosome axes in primary spermatocytes that were more intense on autosomals than on the X (Figure S6). There was a weak CTCF signal in RSs and a clear, cloud-like signal for cohesins (Figure S6). ChIP-seq analysis confirmed these differences in IF signal intensity and density (Figure S6). In fact, we detected 19,347 CTCF ChIP-seq peaks in primary spermatocytes (P/D), with the vast majority (97.1%) being lost in RSs (Figures 5A and 5B).

We then performed ChIP-seq in P/D and RSs on the meiotic cohesins REC8 and RAD21L. In primary spermatocytes (P/D), we detected 11,618 REC8 and 9639 RAD21L peaks distributed across the genome, with substantial overlap (55.8% for RAD21L and 46.3% for REC8) (Figures 5A and 5B). Of the total peaks, 3999 were common to all CTCF (20.6%), RAD21L (41.5%), and REC8 (34.4%). In RSs, we found 11,559 REC8 peaks and 12,507 RAD21L peaks, of which 6383 (51% of RAD21L and 55.2% of REC8) overlapped (Figures 5A and 5B). The vast majority (90.5%) of REC8 peaks observed in primary spermatocytes were maintained in RSs, which contrasted RAD21L, where 40% of the peaks were specific to RSs.

Despite the overlap of RAD21L and REC8 peaks detected genome-wide in P/D, the distribution of REC8 and RAD21L immunolabeling along the axes was not continuous or significantly correlated, as measured by super-resolution microscopy (r^2 = 0.15; Figure S6). The close proximity of meiotic cohesins genome-wide (on average, peaks are scattered every 264.3 Kbp for RAD21L and 219.2 Kbp for REC8) was much lower than the estimated DNA loop size at the axes for mouse pachynema (1.5–2 Mbp; Patel et al., 2019). This pattern suggests that the peaks correspond not only to cohesins loaded at the chromosomal axes (expected every 1.5–2 Mbp), but also to DNA loops out of the axes. Given the close interplay among the organization of chromosomal axes, DNA loops, and the formation of DSBs during early prophase I (Kleckner et al., 2003; Ruiz-Herrera et al., 2017; Wang et al., 2015), we examined the correlation between cohesins and DSBs (SPO11-oligos hotspots; Lange et al., 2016) and H3K4me3 (Brick et al., 2012). Interestingly, both SPO11 hotspots and H3K4me3 marks correlated with cohesin occupancy (STAR Methods; $p < 0.001$), concomitant with open chromatin states. Since DSBs are known to occur genome-wide before being recruited at the chromosomal axes to be repaired (Lange et al., 2016), the observed correlation between SPO11 hotspots and cohesion peaks also suggests the presence of cohesins in DNA loops out of the axes.

Remarkably, we detected a correlation of cohesin genomic distribution with gene expression and local insulation in both primary spermatocytes and RSs (Figures 5 and 6). In P/D, most cohesin peaks (80.7% of RAD21L and 83.3% of REC8) were located in promoter regions (less than 2 kbp from the transcriptional start site [TSS]) of genes with significantly ($p < 0.01$) higher expression than genes without promoter-associated cohesin peaks (Figures 5C–5E). In RSs, cohesin peaks associated with TSSs were reduced to 77.7% for REC8 and 45.5% for RAD21L, suggesting an unequal re-distribution of meiotic cohesins later in spermatogenesis (Figures 5C and 5D). Regardless of RAD21L re-distribution, the overlapping of genes with RAD21L in their promoters between P/D and RSs was statistically significant (p = 0e+00, Fischer's exact test). Despite this reduction of peaks in RSs, genes with cohesin peaks in their TSSs still had significantly higher expression than genes without peaks (Figures 5E and 5F). In contrast, only 25.9% of the CTCF peaks were within promoter regions (i.e., 2 kbp upstream of TSSs) in primary spermatocytes, with the majority located in intergenic regions (Figure 5C). In all cases, CTCF and cohesin peaks were preferentially located (nearly 80% of peaks) in A compartments (permutation test, STAR Methods; $p < 0.001$), consistent with a correlation between chromatin remodeling and active transcription. Although the TAD insulation score was reduced in primary spermatocytes (Figures 3D and 3E), some TADs still remained, with boundaries enriched for CTCF and cohesins (STAR Methods; $p < 0.01$) (i.e., genomic regions with lower TAD insulation scores; Figure 6B) and associated with gene expression (Figure 6C). Meta-border plots of CTCF and cohesin peaks confirmed this trend (Figure 6D). The same pattern was observed in RSs (Figures 6B–6D), suggesting that despite global chromatin remodeling, local insulation is maintained by insulator proteins and can affect gene expression.

Expressed genes with CTCF, RAD21L, and REC8 in their promoters in P/D had GO term enrichments related to protein regulation, modification, and polymerization (e.g., *Usp42*), as well as DNA repair and cellular response to DNA damage stimulus (e.g., *Herc2*), suggesting a regulatory role in spermatogenesis progression. Interestingly, genes with CTCF at their promoters were involved in the transcriptional machinery (e.g., *Nsa2*), whereas genes with both cohesins but not CTCF were essential for posterior neural development of the embryo (e.g., *Cdh2*). In RSs, CTCF was almost absent, but remarkably, when it co-localized with promoters, it was associated with genes implicated in key pathways of embryo development (e.g., *Nanog*). Genes with both cohesins in their promoters were related to the regulation of cell growth and the Wnt signaling pathway (e.g., *Amer3*) and general nervous system development (e.g., *Ccd88a*; Figure 5F). Genes with RS-specific RAD21L peaks had functions involved

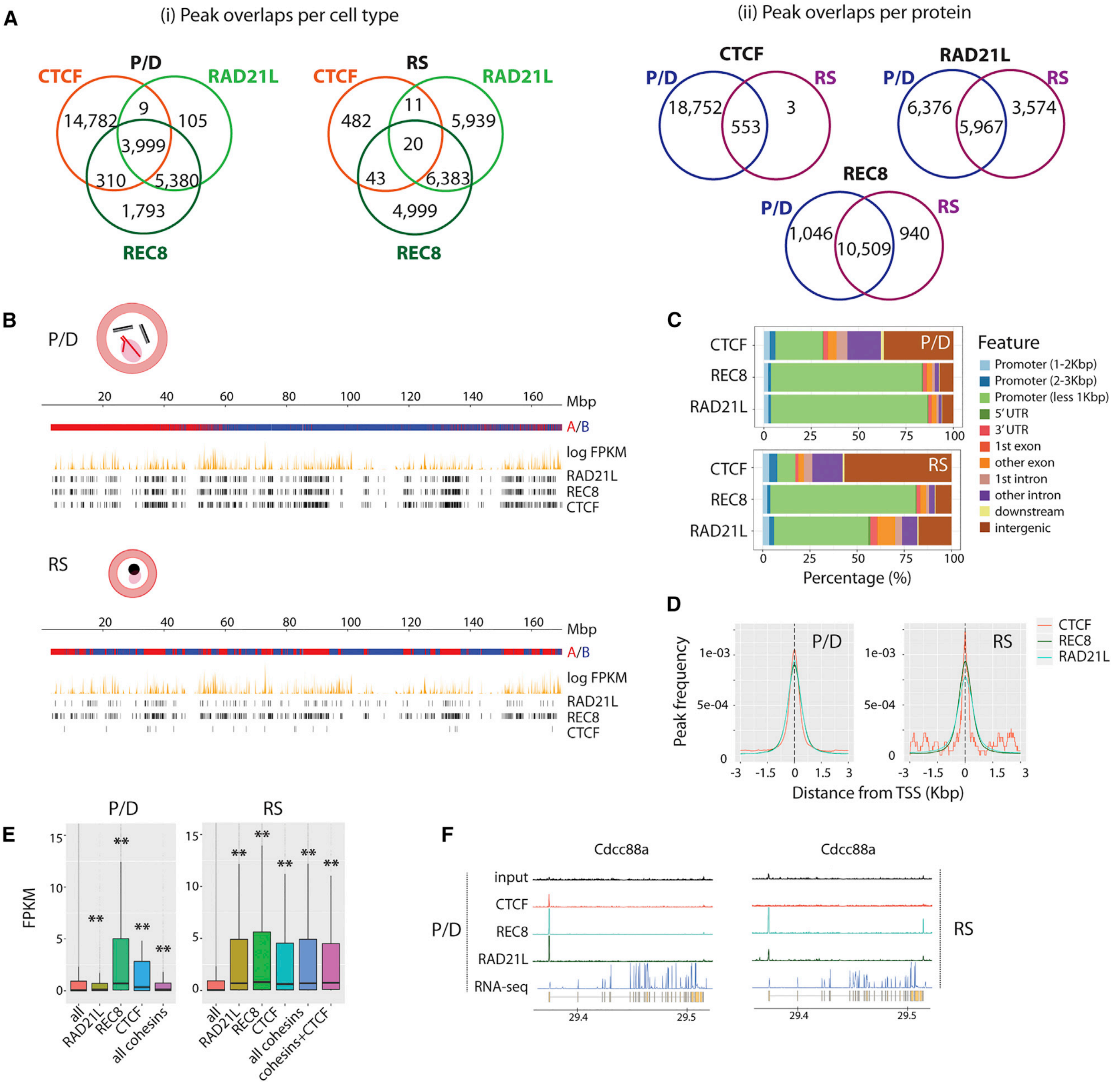

Figure 5. CTCF and Cohesin Profiles in Primary Spermatocytes and RSs

(A) Venn diagrams for CTCF and cohesins in P/D and RSs considering peak overlaps per cell type and peak overlaps per protein.

(B) Representative examples of CTCF and cohesins' genomic distribution along chromosome 1 in P/D and RSs. For each cell type, A-B compartments, gene expression (represented as log FPKM), CTCF peaks, and cohesin peaks are displayed.

(C) Genome-wide distribution of CTCF and cohesin-occupied sites in relation to TSSs and other genomic features in P/D and RSs.

(D) Insulator peak frequencies relative to TSSs of genes in P/D and RSs.

(E) Boxplots representing expression (FPKM values) of genes with CTCF and cohesin peaks located at the TSS. Asterisks represent statistically significant differential gene expression when compared with all genes in the mouse genome ($p < 0.01$).

(F) Examples of CTCF and cohesion-occupied sites in P/D and RSs for the expressed gene Cdc88a.

See also Figure S6.

in oligondendrocyte differentiation and cardiac ventricle development (e.g., *Notch1*), whereas RS-specific REC8 peaks were found to be involved in nervous system development (e.g., *Kif3c*).

Sex Chromosome Silencing Is Coupled with Higher-Order Chromatin Restructuring

The eutherian mammal X chromosome is composed of evolutionary strata that were isolated from recombination with the

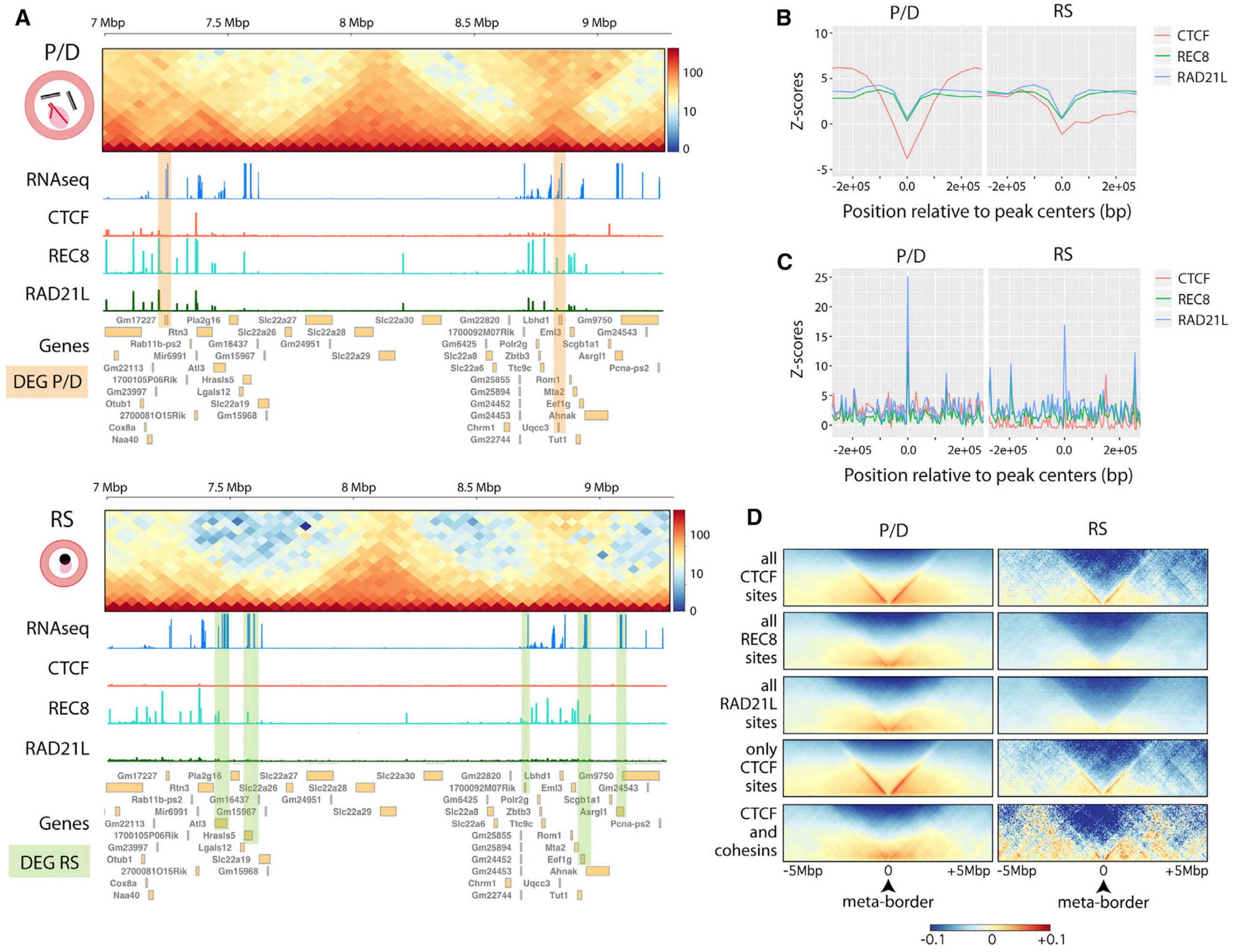

Figure 6. Local Insulation, Cohesin Occupancy, and Gene Expression

(A) Representative examples of CTCF and cohesins' genomic distributions across a specific region of chromosome 19 (from 7 to 9.3 Mbp) in P/D and RSs. For each cell type, Hi-C interaction maps (50-Kbp bins), gene expression (represented as log FPKM), CTCF peaks, cohesin peaks, and genes from NCBI Ref Seq annotation are displayed. Green and orange highlights indicate differentially expressed genes (DEGs) in each cell type: Gm17227 and Uqcc3 for P/D and Atl3; Hrasls5, 1700092M07Rik, Eef1g, and Asrgl1 for RSs.

(B) Distribution of CTCF and cohesin peaks at TAD borders. The y axes represent the TAD insulation Z-score relative to random genomic regions (based on 10,000 permutation tests with randomization, $p < 0.01$).

(C) Distribution of gene expression for CTCF and cohesin peaks located at TAD borders. The y axes represent the TAD insulation Z-score relative to random genomic regions (based on 10,000 permutation tests with randomization, $p < 0.01$).

(D) Meta-plots for all peaks detected in P/D and RSs.

P/D, pachynema-diplonema; RS, round spermatids. See also Figure S6.

Y chromosome at different evolutionary times (Lahn and Page, 1999). In mice, the X chromosome evolutionary strata are highly rearranged, and there are 22 ampliconic regions known to escape MSCI (Mueller et al., 2008) (Figure S7; Table S7). The X chromosome lost its A-B compartment pattern once meiosis was initiated, from L/Z onward (Figures 7A and 7B), consistent with MSCI chromatin remodeling. This translated into an overall reduction in gene expression in P/D compared to Spg, although ampliconic regions were still expressed in P/D, RSs, and sperm (Figure 7C). Importantly, this organization of the X chromosome was maintained in post-meiotic cells (RSs and sperm) (Figures 7A and 7B). In fact, distance-dependent interaction frequencies revealed that genomic interactions at medium genomic distances (1–10 Mbp) were higher in the X chromosome in post-meiotic cells than in autosomes of the same cell type (Figure 2C).

For higher-order chromatin structures at the sub-megabase scale, all chromosomes except X had equivalent TAD dynamics during spermatogenesis. In the X of meiotic and post-meiotic cells, there was a trend for fewer and larger TADs compared to those observed in Spg (Figure 7D; Table S8). The genomic positions of TADs were not equally distributed along the X; in RSs, there were more short TADs detected in the distal regions of

OPEN
ACCESS
CellPress

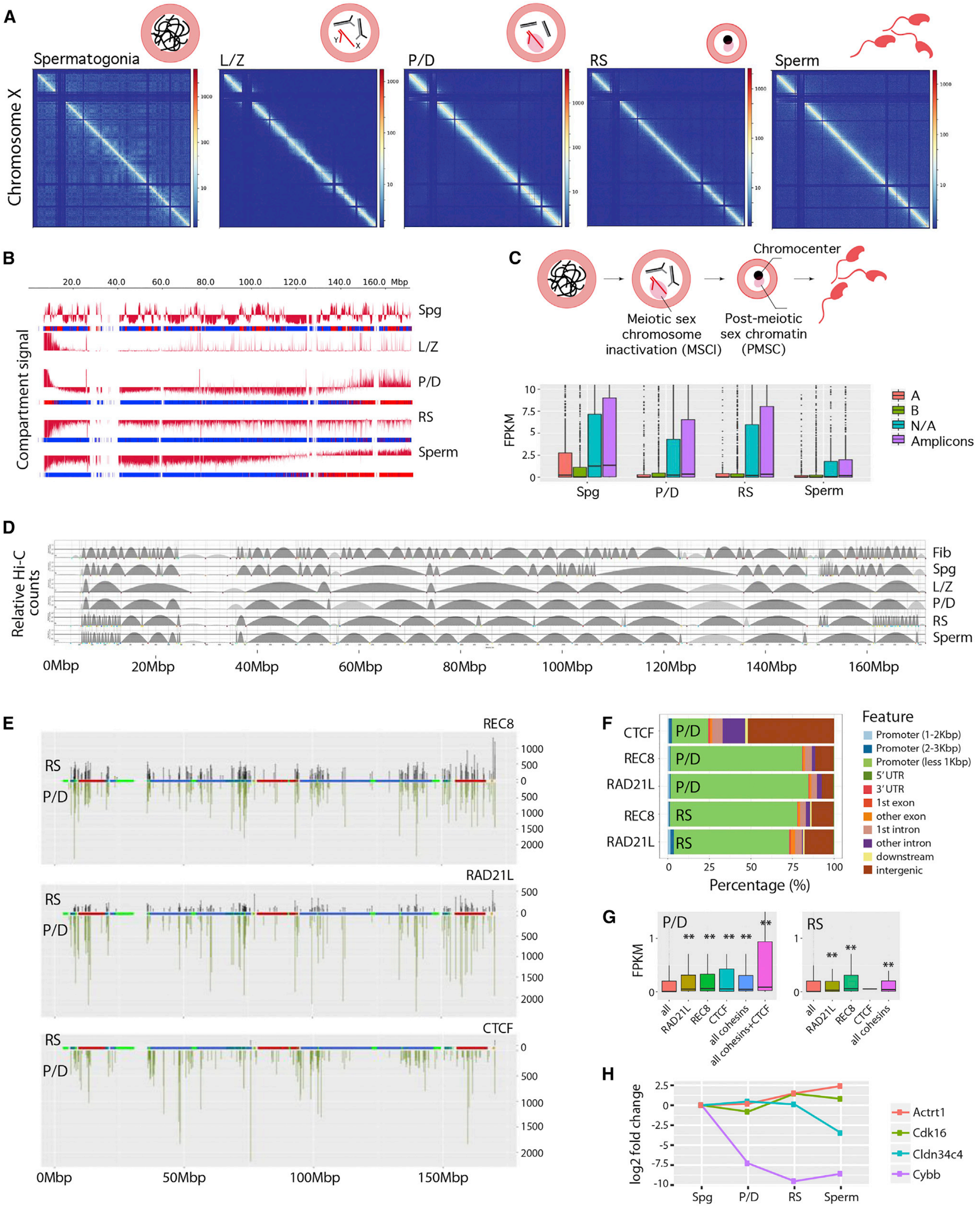

(legend on next page)

the X (Figure 7D), consistent with the increase of autosomal variance of TAD insulation scores in RSs compared to L/Z and P/D (Figure 3E). However, the remainder of the X in RSs maintained the fewer and larger TADs observed in L/Z and P/D. In sperm, the high density of short TADs was only observed in proximal X chromosome long arm (Xq).

Differential CTCF and Cohesin Loading in Autosomes and the X Chromosome

The proportion of cohesin peaks was lower for the X chromosome than for any autosome in both P/D and RSs (Figure 7E). The X represents ~4.9% of the mouse genome, but only accounts for 1.82% of the total RAD21L peaks in P/D and 1.54% of peaks in RSs. Likewise, REC8 peaks on the X chromosome were underrepresented, with 2.12% of the total peaks in P/D and 2.03% of peaks in RSs. The underrepresentation of REC8 ChIP-seq peaks was confirmed by super-resolution microscopy in pachynema, with decreased labeling of REC8 (44.7% ± 10.7%) on the X chromosome axis relative to those of autosomes (Figure S6). In contrast, RAD21L labeling actually increased (25.6% ± 10.6%) on the X (Figure S6).

Overall, 75% of cohesin peaks co-localized with promoter regions (less than 2 kbp upstream of TSSs) in both P/D and RSs in the X chromosome (Figure 7F). These genes had significantly higher expression than genes without promoter-associated cohesin peaks, despite MSCI and PMSC (Figures 7G and 7H), suggesting that genes that escape MSCI correlate with promoter cohesin occupancy. As with the cohesins, CTCF was depleted on the X chromosome relative to the autosomes (1.26% of total peaks were on the X in P/D). However, CTCF was almost completely depleted from the X chromosome in RSs (just two peaks were detected). This was not observed for individual autosomes, on which the proportion of CTCF peaks were generally stable between P/D and RSs. Collectively, these observations point to a novel role for meiotic cohesins in genome organization and function during meiotic prophase I and spermiogenesis.

DISCUSSION

Here, we provide a high-resolution structural and functional atlas of mouse spermatogenesis. Our data reveal the compartmentalization of meiotic chromosomes in both early and late stages of spermatogenesis, which was reflected at different levels: (1) inter- and intra-chromosomal interaction ratios; (2) distance-dependent interaction frequencies; (3) genomic compartments; (4) topological domains; and (5) occupancy of insulator proteins. We provide evidence of a delicate fine-tuning among chromatin remodeling, architectural proteins, and cell-specific gene expression.

Our analyses complement and extend recently published works (Alavattam et al., 2019; Patel et al., 2019; Wang et al., 2019) with a comprehensive view of the sequential developmental stages during mouse spermatogenesis. Pre-meiotic, meiotic, and post-meiotic cells all presented differences in inter- and intra-chromosomal interaction ratios that were distinct from somatic cells (i.e., fibroblasts). Although Spg maintained the equivalent proportion of A-B compartments as fibroblasts, they showed a drastic remodeling of chromosomal compartmentalization. As the first meiotic prophase begins, additional chromatin remodeling appears. The inter- and intra-chromosomal interaction ratio reaches a minimum in primary spermatocytes, with the A-B compartments almost lost in both L/Z and P/D, in contrast to previous observations (Patel et al., 2019). Thus, chromosomal occupancy and compartmentalization inside nuclei were re-arranged (i.e., higher-chromatin structure relaxation) during prophase I, permitting DNA-scaffold assembly and the formation and repair of DSBs, with no distinction between autosomes.

The distance-dependent interaction we detect suggests differences in previously reported mitotic and meiotic chromosome folding (Gibcus et al., 2018; Naumova et al., 2013; Wang et al., 2019). Prophase I cells display two changes in contact probability: the first between 2.5 and 4.5 Mbp and the second at 40 Mbp. This organization can result from two features of chromosome assembly during prophase I. First, the chromatin is anchored as long DNA loops in a protein scaffold composed of specific meiotic cohesins (e.g., REC8 and RAD21L) (Gutiérrez-Caballero et al., 2011; Llano et al., 2012) and proteins of the SC (e.g., SYCP3) (Henderson and Keeney, 2005), preventing interactions below 40 Mbp. Second, the association of recombination hotspots and cohesins in primary spermatocytes suggests that cohesion-mediated transcription in genomic regions out of the

Figure 7. Higher-Order Chromatin Structure and Gene Expression in the X Chromosome

(A) ICE-corrected Hi-C matrices for the X chromosome in mouse germ cells, at a 50-Kbp resolution. Plaid blue regions correspond to non-mapped bins.

(B) Representation of compartment signal (first eigenvector) along the mouse's X chromosome.

(C) Upper panel: Overview of spermatogenesis with two pairs of autosomes (black and gray lines) and the sex chromosomes (X and Y as red lines). Meiotic sex chromosome inactivation (MSCI) characterizes prophase I (shown as a pink cloud; also known as the sex body). In RSs, all centromeres associate in the center of the cell forming the chromocenter, with the X adjacent, forming the post-meiotic sex chromatin (PMSC). Lower panel: Boxplots of expression (FPKM values) of X genes binned according to A-B compartment or ampliconic. N/A are not assigned to a compartment or amplicon.

(D) X chromosome TAD alignments. Arches represent TADs with higher (darker gray) and lower (lighter gray) than expected intra-TAD interactions.

(E) CTCF and cohesin distribution on the X in P/D (green) and RSs (black). Ampliconic regions are green, and evolutionary strata are displayed, with blue representing older strata and red representing newer strata (see Figure S7 for further details).

(F) X chromosome distribution of CTCF and cohesins' occupancy relative to TSSs and other genomic features in P/D and RS.

(G) Expression (FPKM values) of genes with CTCF and cohesin peaks located at promoter regions (−2 kbp from TSS). Asterisks represent statistically significant differential gene expression when compared with all genes in the mouse genome ($p < 0.01$). Boxes represent first and third quartiles, whereas black bars in boxes represent the median values.

(H) Expression changes (versus Spg) of representative X genes that reduce expression in P/D and maintain low levels during spermatogenesis (e.g., Cybb), genes that reduce expression in P/D and increase in RSs and sperm (i.e., *Cdk16*), genes that increase expression in RSs (e.g., *Actr1*), and genes that increase expression in P/D and then reduce (i.e., Cldn34c4).

Fib, fibroblast; Spg, spermatogonia; L/Z, leptonema-zygonema; P/D, pachynema-diplonema; RS, round spermatids. See also Figure S7.

axes can provide an environment conducive to both gene expression and the formation of DSBs. This is reflected by the interactions observed at shorter distances (2.5–4.5 Mbp).

Compartments re-emerged in post-meiotic cells with a remarkable high-level organizational difference between RSs and sperm. Chromosomal territories were re-established in both cell types but with chromatin less densely packed in RSs, as revealed by the interaction ratio and 3D-FISH. In RSs, intra-chromosomal interactions were greater than inter-chromosomal interactions, the opposite to what was observed in sperm. In both cell types, A compartments (which correlate with gene expression) were larger than in pre-meiotic cells and were over-represented when compared to fibroblasts and Spg. Moreover, the transformation of RSs into spermatozoa was accompanied by the transcription of genes related to spermiogenesis and sperm function located in newly created A compartments. This agrees with a second burst of gene expression in RSs (da Cruz et al., 2016) and supports the more recent idea that sperm are not inactive cells (Jodar et al., 2016; Jung et al., 2017). In fact, it was shown that a large number of sperm promoters are in an active epigenetic state, suggesting that this genetic information can influence embryo development upon fertilization (Jung et al., 2017).

There was a remarkable reduction of TADs in sperm. One strength of our approach that differs from others (Jung et al., 2017; Wang et al., 2019) was the cell-sorting strategy, which permitted the isolation of sperm from the rest of the germ cell populations. Coupled with the Hi-C simulations, this permitted us to attribute higher-order chromatin structures specifically to sperm. Thus, the reduction of the TAD insulation score in sperm can be linked to histone replacement by protamines. Protamines replace most histones in sperm, with the help of transition proteins and H2A histone variants, folding DNA into toroidal subunits at the kbp level (Barral et al., 2017). Because of the structural constraints of protamines, it is tempting to speculate that the compact chromatin organization in sperm is associated with the presence of A compartments at the Mbp scale, which likely correlates with active histone marks in sperm (Jung et al., 2017), but not with the formation of fine-scale TAD structures (i.e., kbp).

The genomic atlas for CTCF and meiotic-specific cohesins presented here provides an unprecedented view of the connection among chromatin remodeling, architectural proteins, and gene expression. Strikingly, the vast majority of cohesin peaks detected in primary spermatocytes were localized within promoter regions of genes, with significantly higher expression than non-cohesin-associated genes. The conflicting patterns of RAD21L on the X chromosome, with reduced ChIP peak numbers but increased super-resolution microscopy labeling intensity, are counterintuitive. The increased labeling intensity at the axis suggests that RAD21L has an important X chromosome scaffolding function, but the reduced number of peaks (compared to the autosomes) indicates that the detected peaks correspond to cohesins that do not interact directly with DNA at the axis. In fact, REC8 and RAD21L bind to both head domains of a structural maintenance of chromosome (SMC) heterodimer, forming a ring-like protein structure topologically encircling sister chromatids to the SC, a core axis from which chromatin loops emerge (Haering and Jessberger, 2012). This structure could block REC8 and RAD21L access to chromatin, preventing detection at the axis by ChIP-seq. Our data suggest that cohesins associate with active promoters most probably located in DNA loops out of the axes, hinting at a functional role that adds to the well-known structural role of these cohesins in the formation of meiotic chromosomal axes (Llano et al., 2012). In fact, mice with REC8 and RAD21L deficiencies are infertile; meiosis is arrested in early prophase I (Bannister et al., 2004; Herrán et al., 2011), precluding analysis of potential regulatory roles for cohesins during spermiogenesis.

Our results also point to the role of meiotic cohesins in regulating gene expression after meiosis, which was characterized by the occupancy of RAD21L at the TSSs of expressed genes in RSs. We suggest that CTCF and cohesins could have a synergistic role in establishing transcriptional hubs for early embryonic development in meiotic prophase I (where the three proteins co-localize), while also fine-tuning subsequent spermatogenesis progression. Moreover, cohesins in RSs appear to correlate with the expression of genes implicated in early embryonic development (e.g., *Nanog*), providing immediate access to the major molecular pathways involved in organogenesis upon fertilization. These results suggest that some cellular functional activity is predetermined in early spermiogenesis, before RSs are differentiated into sperm, which raises the intriguing possibility that cohesins play a role in such functional predetermination.

Here, we extend initial observations (Alavattam et al., 2019; Patel et al., 2019; Wang et al., 2019) of X chromosome remodeling in prophase I to pre-meiotic and post-meiotic cells. Our functional and structural analyses show that silencing of the X chromosomes (MSCI) is accompanied by distinct changes in the higher-order chromatin structure at different levels: (1) more intra- than inter-chromosomal interactions, a pattern already present in Spg; (2) strong compartmentalization; and (3) a reduction in both the TAD number and signal once prophase I initiates. Remarkably, architectural proteins were remodeled, which included a reduction of cohesin peaks (compared to autosomes) and an almost complete depletion of CTCF. These data demonstrate that a reduction of the cohesin load correlates with an absence of chromosome X compartmentalization (A/B). Despite MSCI, gene expression still takes place, which correlates with meiotic cohesin occupancy but not with CTCF. Altogether, these observations support a role for cohesin in regulating gene transcription.

In summary, we have implemented a robust workflow to provide an integrated structural and functional framework of the 3D organization of the mouse genome in germ cells. We detected a fine-tuned balance among chromatin remodeling, architectural proteins, and gene expression during spermatogenesis. Overall, our results provide insights into how the structural organization of the genome influences cellular differentiation, especially in the context of the dramatic chromatin changes that take place during the formation and differentiation of the mammalian male germline. Future functional perturbation analyses will help us understand the mechanism by which 3D genome folding changes shape transcriptional activity during spermatogenesis.

STAR★METHODS

Detailed methods are provided in the online version of this paper and include the following:

- KEY RESOURCES TABLE
- LEAD CONTACT AND MATERIALS AVAILABILITY
- EXPERIMENTAL MODEL AND SUBJECT DETAILS
  - Animals
  - Cell lines
- METHOD DETAILS
  - Fluorescence Activated Cell Sorting (FACS) of mouse male germ cells
  - Fibroblast cell culture
  - In nuclei Hi-C
  - Hi-C data processing, binning and normalization
  - Correlation coefficient analysis
  - Averaged contact probability P(s)
  - Inter-/intra-chromosome interaction ratio
  - Inter-subtelomeric interaction quantification
  - Sperm simulations
  - A-B compartments and TAD calling
  - Compartment switching
  - 3D-FISH and confocal microscopy
  - Western blot analysis of CTCF and cohesins in germ cells
  - Spermatocyte spreads and immunofluorescence analysis of CTCF and cohesins
  - Stimulated emission depletion (STED) microscopy
  - ChIP-sequencing
  - ChIP-seq peak calling and annotation
  - RNA-sequencing
  - RNA profiling
  - Analysis of correspondence between compartments and gene expression
  - Local insulation, cohesin occupancy and gene expression
  - X chromosome evolutionary strata and ampliconic regions
- QUANTIFICATION AND STATISTICAL ANALYSIS
- DATA AND CODE AVAILABILITY

SUPPLEMENTAL INFORMATION

Supplemental Information can be found online at https://doi.org/10.1016/j.celrep.2019.06.037.

ACKNOWLEDGMENTS

This work was supported by the Ministry of Economy and Competitiveness (BFU2017-89408-R to A.M.P.; BFU2013-47736-P and BFU2017-85926-P to M.A.M.-R.; and CGL2014-54317-P and CGL2017-83802-P to A.R.-H.) and the Agència de Gestió d'Ajuts Universitaris i de Recerca (AGAUR) (DI2015 to A.R.-H. and R.A.C., as well as SGR468 to M.A.M-R.). Work at CRG, BIST, and UPF was in part funded by the Spanish Ministry of Economy and Competitiveness, 'Centro de Excelencia Severo Ochoa 2013-2017' (SEV-2012-0208), and 'Centro de Excelencia María de Maeztu 2016-2019.' CIC-IBMCC is supported by the Programa de Apoyo a Planes Estratégicos de Investigación de Estructuras de Investigación de Excelencia, co-funded by Junta de Castilla y León (CSI239P18) and the European Regional Development Fund (CLC–2017–01). C.V. is supported by a FPI predoctoral fellowship from the Ministry of Economy and Competitiveness (BES-2015-072924). A.P.-G. is supported by a 'Doctorats Industrials' predoctoral fellowship (AGAUR). H.H. is a Miguel Servet (CP14/00229) researcher funded by the Spanish Institute of Health Carlos III (ISCIII), the Agencia Estatal de Investigación (AEI), and FEDER (SAF2017-89109-P). C.M. is an Asociación Española Contra el Cáncer (AECC) postdoctoral fellow. M.A.M.-R. acknowledges support by the European Research Council under the 7th Framework Program FP7/2007-2013 (ERC grant agreement 609989) and the European Union's Horizon 2020 Research and Innovation Programme (grant agreement 676556). A.M.P. and A.R.-H. also acknowledge support from MeioNet (BFU2015-71786-REDT).

AUTHOR CONTRIBUTIONS

A.R.-H. conceived and devised the study. C.V., A.P.-G., Y.C., F.L.D., H.H., M.A.M.-R., and A.R.-H. designed experiments. C.V., Y.C., F.L.D., F.G., J.S.-C., L.G.-H., E.J., C.M., O.F., and A.R.-H. performed experiments. C.V., A.P.-G., Y.C., F.L.D., L.G.-H., A.M.P., P.D.W., M.A.M.-R., and A.R.-H. analyzed the data. R.A.C., W.S., A.M.P., H.H., M.A.M.-R., and A.R.-H. contributed to reagents and data collection. C.V., A.P.-G., P.D.W., and A.R.-H. wrote the first draft of the manuscript with input from all authors. All authors read and approved the final version of the manuscript.

DECLARATION OF INTERESTS

The authors declare no competing interests.

Received: March 8, 2019
Revised: April 18, 2019
Accepted: June 7, 2019
Published: July 9, 2019

SUPPORTING CITATIONS

The following reference appears in the Supplemental Information: Cortez et al. (2014).

REFERENCES

Alavattam, K.G., Maezawa, S., Sakashita, A., Khoury, H., Barski, A., Kaplan, N., and Namekawa, S.H. (2019). Attenuated chromatin compartmentalization in meiosis and its maturation in sperm development. Nat. Struct. Mol. Biol. *26*, 175–184.

Bannister, L.A., Reinholdt, L.G., Munroe, R.J., and Schimenti, J.C. (2004). Positional cloning and characterization of mouse mei8, a disrupted allelle of the meiotic cohesin Rec8. Genesis *40*, 184–194.

Barral, S., Morozumi, Y., Tanaka, H., Montellier, E., Govin, J., de Dieuleveult, M., Charbonnier, G., Couté, Y., Puthier, D., Buchou, T., et al. (2017). Histone Variant H2A.L.2 Guides Transition Protein-Dependent Protamine Assembly in Male Germ Cells. Mol. Cell *66*, 89–101.e8.

Brick, K., Smagulova, F., Khil, P., Camerini-Otero, R.D., and Petukhova, G.V. (2012). Genetic recombination is directed away from functional genomic elements in mice. Nature *485*, 642–645.

Bushnell, B. (2014). BBMap: A Fast, Accurate, Splice-Aware Aligner. (Department of Energy), Joint Genome Institute.

Carbon, S., Ireland, A., Mungall, C.J., Shu, S., Marshall, B., and Lewis, S.; the AmiGO Hub; the Web Presence Working Group (2008). AmiGO: online access to ontology and annotation data. Bioinformatics *25*, 288–289.

Cortez, D., Marin, R., Toledo-Flores, D., Froidevaux, L., Liechti, A., Waters, P.D., Grützner, F., and Kaessmann, H. (2014). Origins and functional evolution of Y chromosomes across mammals. Nature *508*, 488–493.

da Cruz, I., Rodríguez-Casuriaga, R., Santiñaque, F.F., Farías, J., Curti, G., Capoano, C.A., Folle, G.A., Benavente, R., Sotelo-Silveira, J.R., and Geisinger, A. (2016). Transcriptome analysis of highly purified mouse spermatogenic cell

populations: gene expression signatures switch from meiotic-to postmeiotic-related processes at pachytene stage. BMC Genomics *17*, 294–313.

Dekker, J., Marti-Renom, M.A., and Mirny, L.A. (2013). Exploring the three-dimensional organization of genomes: interpreting chromatin interaction data. Nat. Rev. Genet. *14*, 390–403.

Dixon, J.R., Selvaraj, S., Yue, F., Kim, A., Li, Y., Shen, Y., Hu, M., Liu, J.S., and Ren, B. (2012). Topological domains in mammalian genomes identified by analysis of chromatin interactions. Nature *485*, 376–380.

Dobin, A., Davis, C.A., Schlesinger, F., Drenkow, J., Zaleski, C., Jha, S., Batut, P., Chaisson, M., and Gingeras, T.R. (2013). STAR: ultrafast universal RNA-seq aligner. Bioinformatics *29*, 15–21.

Du, Z., Zheng, H., Huang, B., Ma, R., Wu, J., Zhang, X., He, J., Xiang, Y., Wang, Q., Li, Y., et al. (2017). Allelic reprogramming of 3D chromatin architecture during early mammalian development. Nature *547*, 232–235.

Feng, J., Liu, T., Qin, B., Zhang, Y., and Liu, X.S. (2012). Identifying ChIP-seq enrichment using MACS. Nat. Protoc. *7*, 1728–1740.

Flyamer, I.M., Gassler, J., Imakaev, M., Brandão, H.B., Ulianov, S.V., Abdennur, N., Razin, S.V., Mirny, L.A., and Tachibana-Konwalski, K. (2017). Single-nucleus Hi-C reveals unique chromatin reorganization at oocyte-to-zygote transition. Nature *544*, 110–114.

Gel, B., and Serra, E. (2017). karyoploteR: an R/Bioconductor package to plot customizable genomes displaying arbitrary data. Bioinformatics *33*, 3088–3090.

Gel, B., Díez-Villanueva, A., Serra, E., Buschbeck, M., Peinado, M.A., and Malinverni, R. (2016). regioneR: an R/Bioconductor package for the association analysis of genomic regions based on permutation tests. Bioinformatics *32*, 289–291.

Gibcus, J.H., Samejima, K., Goloborodko, A., Samejima, I., Naumova, N., Nuebler, J., Kanemaki, M.T., Xie, L., Paulson, J.R., Earnshaw, W.C., et al. (2018). A pathway for mitotic chromosome formation. Science *359*, 652.

Gutiérrez-Caballero, C., Herrán, Y., Sánchez-Martín, M., Suja, J.Á., Barbero, J.L., Llano, E., and Pendás, A.M. (2011). Identification and molecular characterization of the mammalian α-kleisin RAD21L. Cell Cycle *10*, 1477–1487.

Haaf, T., and Ward, D.C. (1995). Higher order nuclear structure in mammalian sperm revealed by in situ hybridization and extended chromatin fibers. Exp. Cell Res. *219*, 604–611.

Haering, C.H., and Jessberger, R. (2012). Cohesin in determining chromosome architecture. Exp. Cell Res. *318*, 1386–1393.

Handel, M.A., and Schimenti, J.C. (2010). Genetics of mammalian meiosis: regulation, dynamics and impact on fertility. Nat. Rev. Genet. *11*, 124–136.

Henderson, K.A., and Keeney, S. (2005). Synaptonemal complex formation: where does it start? BioEssays *27*, 995–998.

Herrán, Y., Gutiérrez-Caballero, C., Sánchez-Martín, M., Hernández, T., Viera, A., Barbero, J.L., de Álava, E., de Rooij, D.G., Suja, J.Á., Llano, E., and Pendás, A.M. (2011). The cohesin subunit RAD21L functions in meiotic synapsis and exhibits sexual dimorphism in fertility. EMBO J. *30*, 3091–3105.

Jodar, M., Sendler, E., and Krawetz, S.A. (2016). The protein and transcript profiles of human semen. Cell Tissue Res. *363*, 85–96.

Jung, Y.H., Sauria, M.E.G., Lyu, X., Cheema, M.S., Ausio, J., Taylor, J., and Corces, V.G. (2017). Chromatin states in mouse sperm correlate with embryonic and adult regulatory landscapes. Cell Rep. *18*, 1366–1382.

Keeney, S., Giroux, C.N., and Kleckner, N. (1997). Meiosis-specific DNA double-strand breaks are catalyzed by Spo11, a member of a widely conserved protein family. Cell *88*, 375–384.

Kleckner, N., Storlazzi, A., and Zickler, D. (2003). Coordinate variation in meiotic pachytene SC length and total crossover/chiasma frequency under conditions of constant DNA length. Trends Genet. *19*, 623–628.

Lahn, B.T., and Page, D.C. (1999). Four evolutionary strata on the human X chromosome. Science *286*, 964–967.

Lange, J., Yamada, S., Tischfield, S.E., Pan, J., Kim, S., Zhu, X., Socci, N.D., Jasin, M., and Keeney, S. (2016). The landscape of mouse meiotic double-strand break formation, processing, and repair. Cell *167*, 695–708.e16.

Leng, N., Dawson, J.A., Thomson, J.A., Ruotti, V., Rissman, A.I., Smits, B.M.G., Haag, J.D., Gould, M.N., Stewart, R.M., and Kendziorski, C. (2013). EBSeq: an empirical Bayes hierarchical model for inference in RNA-seq experiments. Bioinformatics *29*, 1035–1043.

Li, H., Handsaker, B., Wysoker, A., Fennell, T., Ruan, J., Homer, N., Marth, G., Abecasis, G., and Durbin, R.; 1000 Genome Project Data Processing Subgroup (2009). The sequence alignment/map format and SAMtools. Bioinformatics *25*, 2078–2079.

Liao, Y., Smyth, G.K., and Shi, W. (2014). featureCounts: an efficient general purpose program for assigning sequence reads to genomic features. Bioinformatics *30*, 923–930.

Lieberman-Aiden, E., van Berkum, N.L., Williams, L., Imakaev, M., Ragoczy, T., Telling, A., Amit, I., Lajoie, B.R., Sabo, P.J., Dorschner, M.O., et al. (2009). Comprehensive mapping of long-range interactions reveals folding principles of the human genome. Science *326*, 289–293.

Llano, E., Herrán, Y., García-Tuñón, I., Gutiérrez-Caballero, C., de Álava, E., Barbero, J.L., Schimenti, J., de Rooij, D.G., Sánchez-Martín, M., and Pendás, A.M. (2012). Meiotic cohesin complexes are essential for the formation of the axial element in mice. J. Cell Biol. *197*, 877–885.

Love, M.I., Huber, W., and Anders, S. (2014). Moderated estimation of fold change and dispersion for RNA-seq data with DESeq2. Genome Biol. *15*, 550–571.

Marco-Sola, S., Sammeth, M., Guigó, R., and Ribeca, P. (2012). The GEM mapper: fast, accurate and versatile alignment by filtration. Nat. Methods *9*, 1185–1188.

Mi, H., Huang, X., Muruganujan, A., Tang, H., Mills, C., Kang, D., and Thomas, P.D. (2017). PANTHER version 11: expanded annotation data from Gene Ontology and Reactome pathways, and data analysis tool enhancements. Nucleic Acids Res. *45*, D183–D189.

Mueller, J.L., Mahadevaiah, S.K., Park, P.J., Warburton, P.E., Page, D.C., and Turner, J.M.A. (2008). The mouse X chromosome is enriched for multicopy testis genes showing postmeiotic expression. Nat. Genet. *40*, 794–799.

Namekawa, S.H., Park, P.J., Zhang, L.-F., Shima, J.E., McCarrey, J.R., Griswold, M.D., and Lee, J.T. (2006). Postmeiotic sex chromatin in the male germline of mice. Curr. Biol. *16*, 660–667.

Naumova, N., Imakaev, M., Fudenberg, G., Zhan, Y., Lajoie, B.R., Mirny, L.A., and Dekker, J. (2013). Organization of the mitotic chromosome. Science *342*, 948–953.

Patel, L., Kang, R., Rosenberg, S.C., Qiu, Y., Raviram, R., Chee, S., Hu, R., Ren, B., Cole, F., and Corbett, K.D. (2019). Dynamic reorganization of the genome shapes the recombination landscape in meiotic prophase. Nat. Struct. Mol. Biol. *26*, 164–174.

Picelli, S., Faridani, O.R., Björklund, A.K., Winberg, G., Sagasser, S., and Sandberg, R. (2014). Full-length RNA-seq from single cells using Smart-seq2. Nat. Protoc. *9*, 171–181.

Quinlan, A.R., and Hall, I.M. (2010). BEDTools: a flexible suite of utilities for comparing genomic features. Bioinformatics *26*, 841–842.

Ramírez, F., Ryan, D.P., Grüning, B., Bhardwaj, V., Kilpert, F., Richter, A.S., Heyne, S., Dündar, F., and Manke, T. (2016). deepTools2: a next generation web server for deep-sequencing data analysis. Nucleic Acids Res. *44* (*W1*), W160–W165.

Ramírez, F., Bhardwaj, V., Arrigoni, L., Lam, K.C., Grüning, B.A., Villaveces, J., Habermann, B., Akhtar, A., and Manke, T. (2018). High-resolution TADs reveal DNA sequences underlying genome organization in flies. Nature Communications *9*, 189.

Rao, S.S.P., Huntley, M.H., Durand, N.C., Stamenova, E.K., Bochkov, I.D., Robinson, J.T., Sanborn, A.L., Machol, I., Omer, A.D., Lander, E.S., and Aiden, E.L. (2014). A 3D map of the human genome at kilobase resolution reveals principles of chromatin looping. Cell *159*, 1665–1680.

Rau, A., Gallopin, M., Celeux, G., and Jaffrézic, F. (2013). Data-based filtering for replicated high-throughput transcriptome sequencing experiments. Bioinformatics *29*, 2146–2152.

Reig-Viader, R., Garcia-Caldés, M., and Ruiz-Herrera, A. (2016). Telomere homeostasis in mammalian germ cells: a review. Chromosoma *125*, 337–351.

Robinson, M.D., McCarthy, D.J., and Smyth, G.K. (2010). edgeR: a Bioconductor package for differential expression analysis of digital gene expression data. Bioinformatics *26*, 139–140.

Ruiz-Herrera, A., Vozdova, M., Fernández, J., Sebestova, H., Capilla, L., Frohlich, J., Vara, C., Hernández-Marsal, A., Sipek, J., Robinson, T.J., and Rubes, J. (2017). Recombination correlates with synaptonemal complex length and chromatin loop size in bovids-insights into mammalian meiotic chromosomal organization. Chromosoma *126*, 615–631.

Sánchez-Guillén, R.A., Capilla, L., Reig-Viader, R., Martínez-Plana, M., Pardo-Camacho, C., Andrés-Nieto, M., Ventura, J., and Ruiz-Herrera, A. (2015). On the origin of Robertsonian fusions in nature: evidence of telomere shortening in wild house mice. J. Evol. Biol. *28*, 241–249.

Sassone-Corsi, P. (2002). Unique chromatin remodeling and transcriptional regulation in spermatogenesis. Science *296*, 2176–2178.

Serra, F., Baù, D., Goodstadt, M., Castillo, D., Filion, G.J., and Marti-Renom, M.A. (2017). Automatic analysis and 3D-modelling of Hi-C data using TADbit reveals structural features of the fly chromatin colors. PLoS Comput. Biol. *13*, e1005665.

Tarazona, S., García-Alcalde, F., Dopazo, J., Ferrer, A., and Conesa, A. (2011). Differential expression in RNA-seq: a matter of depth. Genome Res. *21*, 2213–2223.

Thorvaldsdóttir, H., Robinson, J.T., and Mesirov, J.P. (2013). Integrative Genomics Viewer (IGV): high-performance genomics data visualization and exploration. Brief. Bioinform. *14*, 178–192.

Tian, T., Liu, Y., Yan, H., You, Q., Yi, X., Du, Z., Xu, W., and Su, Z. (2017). agriGO v2.0: a GO analysis toolkit for the agricultural community, 2017 update. Nucleic Acids Res. *45*, W122–W129.

Turner, J.M.A. (2007). Meiotic sex chromosome inactivation. Development *134*, 1823–1831.

Wang, S., Zickler, D., Kleckner, N., and Zhang, L. (2015). Meiotic crossover patterns: obligatory crossover, interference and homeostasis in a single process. Cell Cycle *14*, 305–314.

Wang, Y., Wang, H., Zhang, Y., Du, Z., Si, W., Fan, S., Qin, D., Wang, M., Duan, Y., Li, L., et al. (2019). Reprogramming of meiotic chromatin architecture during spermatogenesis. Mol. Cell *73*, 547–561.e6.

Wickham, H. (2016). ggplot2: Elegant Graphics for Data Analysis (Springer-Verlag).

Yang, T., Zhang, F., Yardımcı, G.G., Song, F., Hardison, R.C., Noble, W.S., Yue, F., and Li, Q. (2017). HiCRep: assessing the reproducibility of Hi-C data using a stratum-adjusted correlation coefficient. Genome Res. *27*, 1939–1949.

Yu, G., Wang, L.G., and He, Q.Y. (2015). ChIPseeker: an R/Bioconductor package for ChIP peak annotation, comparison and visualization. Bioinformatics *31*, 2382–2383.

Zhu, L.J., Gazin, C., Lawson, N.D., Pagès, H., Lin, S.M., Lapointe, D.S., and Green, M.R. (2010). ChIPpeakAnno: a Bioconductor package to annotate ChIP-seq and ChIP-chip data. BMC Bioinformatics *11*, 237–247.

STAR★METHODS

KEY RESOURCES TABLE

Reagent or Resource	Source	Identifier
Antibodies		
Anti-mouse SYCP3	Abcam	Cat#ab97672; RRID:AB_10678841
Anti-Rabbit DMC1	Santa Cruz Biotechnologies	Cat#sc-22768; RRID:AB_2277191
Anti-mouse Cy5	Jackson Immunoresearch	Cat#115-175-146; RRID:AB_2338713
Anti-rabbit FITC	Jackson Immunoresearch	Cat#111-095-003; RRID:AB_2337972
Anti-CTCF	Millipore	Cat#07-729; RRID:AB_441965
Anti-REC8	Courtesy of A.M. Pendás	N/A
Anti-RAD21L	Courtesy of A.M. Pendás	N/A
Anti-HRP-PO	Bio-Rad	Cat#1706515; RRID:AB_11125142
Chemicals, Peptides and Recombinant Proteins		
Collagenase Type II	Life Technologies	#17101015
DNase I	Sigma-Aldrich	#DN25-10MG
Trypsin from bovine pancreas	Sigma-Aldrich	#T9935-100MG
Hoechst 33342	Life Technologies	#H3570-10ml
Complete Protease Inhibitor	Roche	#1187358001
MboI	New England Biolabs	#R0147M
Biotin-14-dATP	Life Technologies	#19524-016
DNA Polymerase I, large (Klenow) Fragment	New England Biolabs	#M0210M
T4 DNA Ligase	New England Biolabs	#M0202M
Dynabeads MyOne Streptavidin T1	Life Technologies	#65001
T4 Polunucleotides Kinase	New England Biolabs	#M0201L
T4 DNA Polymerase	New England Biolabs	#M0212M
Klenow Fragment 3′- > 5′ exo-	New England Biolabs	#M0212M
AMPure XP Beads	Beckman-Coulter	#A63880
2x Laemmli Buffer	Bio-Rad Laboratories	#1610737
Trans-Blot® Turbo Transfer Packs	Bio-Rad Laboratories	#1704158
Ponceau S solution	Sigma-Aldrich	#P7170-1L
Clarity™ Western ECL Substrate, 500 ml	Bio-Rad Laboratories	#1705061
OneDay ChIP kit	Diagenode	C01010080
Unblocked protein A	Diagenode	C03020002
NEBNext® End Repair Module	New England Biolabs	E6050S
NEBNext® End Repair Reaction Buffer	New England Biolabs	B6052S
NEBNEXT® dA-tailing Reaction Buffer	New England Biolabs	B6059S
Deposited Data		
Hi-C data	This paper	GEO:GSE132054
ChIP-seq data	This paper	GEO:GSE132054
RNA-seq data	This paper	GEO:GSE132054
SPO11-oligos hotspots	Lange et al., 2016	GEO:GSE84689
H3K4me3 data	Brick et al., 2012	GEO:GSE35498
Hi-C data from sperm	Jung et al., 2017	SRR3225862 and SRR3225863
Experimental Models: Organisms/Strains		
C57BL/6J	Charles River Laboratories	N/A

(*Continued on next page*)

Continued

Reagent or Resource	Source	Identifier
Software and algorithms		
BBDuk (version 10/2015)	Bushnell, 2014	https://sourceforge.net/projects/bbmap/
TADbit (version 0.2.0.23)	Serra et al. (2017	https://github.com/3DGenomes/TADbit
GEM (version 1.7.1)	Marco-Sola et al., 2012	https://sourceforge.net/projects/gemlibrary/
HiCExplorer (version 1.8.1)	Ramírez et al., 2018	https://github.com/deeptools/HiCExplorer
HiCRep (version 1.4)	Yang et al., 2017	https://github.com/MonkeyLB/hicrep
BEDtools (version 2.17)	Quinlan and Hall, 2010	https://github.com/arq5x/bedtools2
SAM tools	Li et al., 2009	http://samtools.sourceforge.net/
Deep tools	Ramírez et al., 2016	https://github.com/deeptools/deepTools
MACS2	Feng et al., 2012	https://github.com/taoliu/MACS
IGV tools	Thorvaldsdóttir et al., 2013	https://igv.org/
ChIPseeker	Yu et al., 2015	https://github.com/GuangchuangYu/ChIPseeker
KaryoploteR	Gel and Serra, 2017	http://bioconductor.org/packages/release/bioc/html/karyoploteR.html
RegioneR	Gel et al., 2016	https://bioconductor.org/packages/release/bioc/html/regioneR.html
Panther	Mi et al., 2017	http://www.pantherdb.org/
AmiGo	Carbon et al., 2008	http://amigo.geneontology.org
AIR	This paper	https://transcriptomics.cloud

LEAD CONTACT AND MATERIALS AVAILABILITY

Further information and requests for resources and reagents should be directed to, and will be fulfilled by, the Lead Contact: Aurora Ruiz-Herrera (aurora.ruizherrera@uab.cat).

EXPERIMENTAL MODEL AND SUBJECT DETAILS

Animals

C57BL/6J (B6) male mice at 8-17 weeks of age were purchased from the Charles River Laboratories. Animal maintenance and experimental procedures were carried out according to the Ethics Committee on Animal and Human Experimentation (CEEAH) guidelines from Universitat Autònoma de Barcelona (UAB).

Cell lines

A primary fibroblast cell line derived form a male mouse (C57BL/6J strain) previously established in our lab (Sánchez-Guillén et al., 2015), and cultured in DMEM medium supplemented with 10% fetal bovine serum and 1% PenStrep at 37°C and 5% CO_2.

METHOD DETAILS

Fluorescence Activated Cell Sorting (FACS) of mouse male germ cells

Male mice were dissected by an inguinal incision to remove whole testis and cauda epididymis. Sperm cells were recovered from the epididymis by pressing the deferent tube gently. Both decapsulated testis and epididymis were mechanically disaggregated together with the ejaculate, incubated in GBSS with collagenase type II (Life Technologies) (0.5 mg/ml) and DNase I (Sigma Aldrich) (1 ng/μl) at 33°C for 15min with agitation. Fetal bovine serum (ThermoFisher Scientific) was added to the mix at a proportion of 5% in order to inactivate trypsin. The cell suspension was filtered through a 70 μm diameter cell strainer and subsequently centrifuged for 3 min at 1,800 xg. Cells were then dyed with 5 μg/ml of Hoechst 33342 (Life Technologies) for 30 min at 33°C with agitation, then held at 4°C until sorting. Germ cells were sorted using a BD InfluxTM (BD Biosciences) coupled with an ultraviolet laser (355 nm). Subsequently, three sorting strategies were followed in order to obtain a total of six highly enriched germ cell populations: (i) spermatogonia (2n, 2c), (ii) spermatocytes at leptonema/zygonema (L/Z) stage (2n, 4c), (iii) spermatocytes at pachynema/diplonema (P/D) stage (2n, 4c), (iv) secondary spermatocytes (1n, 2c), (v) round spermatids and (vi) sperm.

Strategy #1: Four main germ cell populations (spermatogonia, P/D, secondary spermatocytes and round spermatids) were first isolated by plotting Hoechst Blue (UV355-460/50) versus Hoechst red (UV355-670/30) emissions to discriminate cells by both their DNA content and their complexity (Figure S1A). The spermatogonia fraction (2n, 2c) represented a heterogeneous population of undifferentiated and differentiating spermatogonia.

Strategy #2: In order to discriminate primary spermatocytes in L/Z and P/D stages, an "in solution" immunofluorescence using DMC1 (1:300, Santa Cruz Biotechnologies) and SYCP3 (1:1000, Abcam) antibodies was performed before sorting. Briefly, DMC1 (1:300, Santa Cruz Biotechnologies) and SYCP3 (1:1000, Abcam) antibodies were added to the cell suspension that resulted from testis disaggregation. Cells showing DMC1(+)/SYCP3(+) staining corresponded to primary spermatocytes in L/Z stage, whereas cells at P/D stage appeared with DMC1(-)/SYCP3(+) staining (Figure S1B).

Strategy #3: A third approach was followed to obtain highly enriched populations of sperm and round spermatids as both cell types have the same DNA content and can only be discriminated by morphology. We followed a re-gating approach since cell morphology can be discriminated in the flow sorter by how cells project a side shadow (side scatter, SSC) and a forward shadow (forward scatter, FSC), a plot for each initial gate was drawn (SSC versus FSC plot). With this approach, and applying a restrictive re-gating approach, sperm and round spermatids were isolated with high enrichment (Figure S1C). This allowed us to exclude elongating spermatids from these samples. Likewise, isolation of sperm from cauda epididymis was conducted by re-gating of the c fraction, resulting in isolated sperm Figure S1D).

Irrespective of the sorting strategy, all cell populations were collected after sorting in 1x PBS, centrifuged for 5 min at 1,800 xg. The supernatant was discarded, and cell pellets were flash-frozen at −80°C until use. Sorts were typically 3-6 hours to collect between 0.2×10^6 and 3.2×10^6 cells, depending on the germ cell population.

Cell enrichment of each flow-sorted population was evaluated by immunofluorescence using specific meiotic proteins and DAPI morphology (Figure S1). Spermatogonia were distinguished using an anti-CD90 (Thy-1+) (1:10) antibody and DAPI morphology. For primary spermatocytes, prophase-I stages (leptonema, zygonema, pachynema and diplonema) were identified based on SYCP3 (1:400) and ɣH2AX patterns (1:300). For secondary spermatocytes, immunofluorescence using anti-H3K9me3 (1:500), anti-actin (1:400) and anti-tubulin (1:1000) was performed. Cell enrichment of round spermatids and sperm fractions was determined based on nucleus morphology and DAPI pattern. Cells were fixed on slides and then mounted with DAPI diluted in Vectashield (Vector Laboratories). In all cases, slides were analyzed using a fluorescence microscopy (Axiophot, Zeiss) coupled with a ProgRes ® CS10plus, Jenoptik camera. Representative images were captured with ACO XY (A. Coloma, Open Microscopy). Between 50 and 100 cells were counted for each flow-sorted population. Only sorted populations with enrichment above 80% were considered for subsequent experiments.

Fibroblast cell culture

The mouse primary fibroblast cell line was cultured to 100% confluence. A cell fraction was kept for quality controls (see below) and the rest were fixated accordingly to the crosslink step of the Hi-C method (see *in nuclei* Hi-C section) for adherent cells.

Quality controls consisted of generating a modal karyotype and flow cytometry cell cycle analysis. Chromosome spreads were obtained using standard protocols. Briefly, cells were arrested in metaphase by adding 80 μL of Colcemid (10 μg/ml) (Sigma) to 10ml of medium for 2h and then trypsinised. Cells were spun down at 600 xg for 7 minutes and resuspended in 5ml of hypotonic solution (0.075M KCl) for 20 minutes at 37°C. Chromosomes were then fixed by addition of fixative solution (3:1 methanol/acetic acid) and metaphase spreads were obtained by dropping 15 μl of cell suspension onto a cleaned dry slide. Slides were kept at −20°C until use. Metaphases were stained homogenously with Giemsa solution for analysis of the modal karyotype. An optical microscope (model Zeiss Axioskop) equipped with a charged coupled device camera (ProgResR CS10Plus, Jenoptik) was used for the microscope analysis. Good-quality metaphases were captured with the program Progress Capture 2.7.7 and analyzed for each specimen, obtaining the modal karyotype.

For the cell cycle analysis, fibroblasts were cultured until cells reached 100% confluence. Next, cells were fixated with 1% formaldehyde for 10' at RT and incubated with glycine 0.125M for 5′ at RT and for 15' at 4°C to stop the crosslinking reaction. Subsequently, cells were scrapped off the flask, pelleted and resuspended in 1x PBS to finally be stained with Hoechst 33342. Cell cycle was analyzed using a BD InfluxTM (BD Biosciences) coupled with an ultraviolet laser (355 nm) to reveal that over 74% of the cells were in G1-phase (Figure S1).

In nuclei Hi-C

The generation of Hi-C libraries was optimized from the protocol developed by Rao et al. (2014). Different approaches were used depending on the cell type analyzed (somatic and germ cells, see below). Two replicates for each FACS-sorted population were obtained (with the exception of primary spermatocytes at L/Z stage where one single experiment was performed) from a total of 7.7×10^6 spermatogonia, 1.7×10^6 primary spermatocytes at L/Z stage, 20.9×10^6 primary spermatocytes at P/D stage, 15.7×10^6 secondary spermatocytes, 107.7×10^6 round spermatids and 113.8×10^6 sperm.

Somatic cells: Fibroblasts were grown until reaching confluence in supplemented DMEM medium. Cells were then washed with PBS and fixed with 1% formaldehyde for 10' at RT. Cells were then incubated with glycine 0.125M for 5' at RT and for 15' at 4°C to stop the crosslinking reaction. Following, 2 mL Trypsin (Fischer Scientific) were added, and cells were incubated for 8' at RT and then washed twice with PBS. Cells were scraped and collected in a tube and centrifuged for 5 minutes at a maximum speed of 1,800 xg.

Germ cells: Germ cells at a concentration of 1 million per 500 μl were incubated in formaldehyde (1%) for 10 min with agitation prior to FACS. Glycine (Sigma Aldrich) was added at a final concentration of 0.125 M and incubated with agitation at room temperature for

5 min and then at 4°C for 15 min. Cells were then centrifuged for 10 min at 290 xg at 4°C and resuspended in 3 mL 1x PBS in case of direct Hoechst staining, or in the according volume of block solution if immunofluorescence was the following step.

Each crosslinked cell aliquot was resuspended with lysis buffer and incubated on ice for 30' and then centrifuged for 5′ at 1,800 xg. Pellets were washed with 1x NEB2 buffer (twice) and resuspended with NEB2 buffer with 10% SDS at RT and incubated for 10' at 65°C with agitation (300 rpm). NEB2 buffer with 10% Triton X-100 solution was added and cells were incubated for 30' at 37°C. Following, cells were centrifuged for 5′ at 1,800 xg (4°C) and washed with 1x NEB2 buffer twice. An aliquot for ND (Non-Digested) control was taken from the sample to be processed and incubated at 37°C together with the digested sample. 400 U of MboI were added to the rest of the samples and chromatin was digested O/N at 37°C with agitation. An aliquot from the digested samples was taken for digestion controls. The full samples were kept at 37°C while the digestion control was performed. Proteinase K (10 mg/ml) (ThermoFisher) was added and the aliquoted samples were incubated for 45'-60' at 65°C followed by Phenol:Chloroform purification. The quality of the sample was checked by running on an 0.8% agarose gel.

After digestion, a small aliquot from the samples was kept as a Non-Ligated control and another aliquot was directly ligated without reparation (ligation control). Samples were centrifuged for 5′ at 1,800 xg. Samples were then washed with 1x NEB2 buffer, twice. After the second wash, samples were directly resuspended with the reparation mix (1x NEB2 buffer, 0.05 mM dCTP, 0.05 mM dTTP, 0.05 mM, 50 mM biotin-dATP (ThermoFisher), 50U Kleenow (NEB, M02010M), H_2O). Samples were incubated for 45' at 37°C and for 10' at 65°C and centrifuged for 5′ at 1,800 xg and then resuspended with ligation buffer [1x NEB T4 ligase buffer, 10% Triton x-100, 0.1 mg/ml BSA, 5 μl (10000 U cohesive) ligase (2000 U/ μl) (NEB, M0201M), 963 μl H_2O]. Samples were incubated at 16°C for at least 4h or O/N with mixing, then centrifuged for 5′ at 1,800 xg and resuspended in 1x NEB2 buffer. Samples were incubated with RNaseA (10 mg/ml) (ThermoFisher) for 15 minutes at 37°C. The mix was incubated with Proteinase K (10 mg/ml) at 65°C O/N to reverse the cross-link. Samples were cooled to RT and purified with Phenol/Chloroform/Isoamyl alcohol. DNA content was measured on a Qubit. Samples were sonicated: 20 s time ON, 60 s time OFF, 8 cycles. Samples were then loaded in an electrophoresis gel of 1.2% agarose to check fragment size.

Samples were incubated for 30' with rotation at RT with Dynabeads MyOne Streptavidin T1 beads (Life Technologies) and 2x Binding Buffer (10 mM TrisHCl, 1mM EDTA, 2M NaCl). Beads were washed twice with Binding Buffer and resuspended in the end repair mix [1x NEB T4 DNA ligase buffer with 10 mM ATP, 25 mM dNTP mix, 10U/μl NEB T4 PNK (NEB M0201), 3U/μl T4 DNA polymerase I (NEB M0203), 5U/μl NEB DNA polymerase I (Klenow) (NEB 0210)]. Samples were incubated for 30' at RT. Beads were washed with 1x Binding Buffer, twice. Beads were subsequently resuspended in the dATP attachment master mix (1x NEBuffer 2, 0.5mM dATP, 5U/μl NEB Klenow exo minus (NEB, M0212), H_2O). Samples were incubated at 37°C for 30'. The beads were washed with 1x Binding Buffer, twice and resuspended with 1x NEB Quick ligation buffer (NEB, B6058).

NEB T4 DNA ligase and Illumina indexed adapters were added and thoroughly mixed. Samples were incubated for 15' at RT, beads were captured, and the supernatant was discarded. Beads were then washed with 1x Binding Buffer, twice. The beads were resuspended in Tris buffer (Elution buffer). PCR (5μl beads + 5.5 μl H2O, 1.25 μl primer P5 25 mM, 1.25 μl primer P7 25 mM, 12,5 cocktail master mix NEB) was performed (98°C 30 s, 98°C 10 s, 60°C 30 s x 8 cycles, 72°C 30 s, 72°C 5′). After PCR, beads were captured on a magnet and the PCR was transferred to a new tube. The library was quantified using a Qubit fluorometer. 5μl from the PCR products were run in a 1.2% agarose electrophoresis gel to confirm range of sizes. A 1:1 amount of AmpureBeads (Beckman Coulter) was added to the samples and incubated for 10' at RT. The beads were washed twice with 70% ethanol without mixing. The beads were then eluded and incubated at RT, for 5′. AmpureBeads were separated on a magnet, and the solution was transferred to a fresh tube. 5μl of each sample was loaded to an electrophoresis gel of 1.2% agarose for a final size check. DNA quantity was measured with the Qubit fluorometer. Libraries were submitted for Illumina sequencing (paired-end 75bp each side on HiSeq 2500, v4).

Hi-C data processing, binning and normalization

Quality check and trimming were conducted using BBDuk (version 10/2015) (Bushnell, 2014). Setting a minimum read length of 35 bp and a minimum Phred quality score of 20, adapters and low-quality reads were removed while preserving their longest high-quality regions. Then, reads were processed with TADbit (version 0.2.0.23) (Serra et al., 2017), which makes use of the GEM (version 1.7.1) mapper (Marco-Sola et al., 2012) to iteratively map them against the mouse genome (version mm10). Reads were mapped from 15 bp toward using a step size of 5 bp. The filters used in order to remove possible artifacts were the following: "self-circle," "dangling-end," "error," "extra dangling-end," "too short," "too large," "duplicated," and "random breaks." The maximum molecule length parameter was set at 2 times the 99.9 percentile of the insert size distribution, returned by the "insert_size" from TADbit. The maximum distance of a read to a cleavage site was set to the 99.9 percentile of the insert size distribution.

An in-house script was used for binning and data normalization. This script imported the "HiC_data" module of TADbit, read the map files generated after the artifacts filtering step, binned the reads into a square matrix of 50 Kbp, and stored the matrix into a file in NPZ format (raw matrix). Afterward, HiCExplorer (version 1.8.1) (Ramírez et al., 2018) was used to normalize with the ICE (Iterative Correction and Eigenvector decomposition).

Correlation coefficient analysis

Pairwise comparisons between biological replicates were performed using HiCRep (version 1.4) (Yang et al., 2017), under a smoothing parameter of 5 and a considered distance over 10 Mbp. Since HiCRep only handles intra-chromosome raw matrices, each pairwise comparison yielded 20 correlation scores (19 autosome chromosomes + sex chromosome X). The Y chromosome was

excluded from the analysis due to the lower number of interactions detected in our analysis (less than 1% of the overall detected interactions) and the highly repetitive nature of this chromosome. The correlation between 2 replicates was defined as the mean of the 20 correlation scores.

Averaged contact probability P(s)

ICE-normalized matrices were scaled with a factor of 1/sum(matrix). The resulting matrices were then input to "hicPlotDistVsCounts" from the HiCExplorer package in order to obtain the contact probability P(s).

Inter-/intra-chromosome interaction ratio

ICE-normalized data stored in matrices were exported with HiCExplorer to the GInteractions format, which consists of 7 columns: chromosome, start and end from bin 1, chromosome, start and end from bin 2, and the amount of interaction. The GInteractions tables were imported in R for further quantification of intra-chromosome and intra-chromosome interactions and plotting.

Inter-subtelomeric interaction quantification

ICE-normalized matrices were scaled with a factor of 1,000,000/sum(matrix) and exported with HiCExplorer to GInteractions format. The GInteractions tables were imported in R for this inter-telomere interaction quantification. Since the telomeric and centromeric regions (annotated from the beginning of each chromosome to 2.9 Mbp according to the UCSC Table Browser) were masked due to the low-count filtering step prior to ICE normalization, we only considered inter-chromosome interactions between loci located within genomic positions 3 to 3.5 Mbp in each chromosome. Differences in the subtelomeric interaction frequencies between cell types were assessed with the Wilcoxon test.

Sperm simulations

In order to validate our enriched sperm population, we simulated six Hi-C sperm datasets of 100×10^6 reads with different proportions, from 0 to 100% by steps of 20%, of fibroblast reads. Both sperm and fibroblasts reads were derived from our generated libraries. Previously published data on sperm (SRR3225862 and SRR3225863 accessions from Jung et al. (2017) were also downloaded. These datasets underwent a quality check, Hi-C data processing, binning and normalization steps as described above. The resulting raw Hi-C matrices were used for correlation coefficient analysis while the ICE-normalized matrices were used to calculate the averaged contact probability P(s) (Figure S5).

A-B compartments and TAD calling

Raw matrices were used for the definition of A-B compartments. Columns with a low number of counts were filtered out using TADbit, setting the parameter min_count to 10. Since TADbit fits the column count distribution into a polynomial distribution, columns with a number of counts smaller than the first antimode of the distribution, which cannot be smaller than the min_count parameter, are filtered out. Then, the genome-wide matrices were normalized by the expected interactions at a given distance and by visibility by means of one-iteration of the ICE method. The correlation analysis was also performed with TADbit. In-house scripts computed A-B compartments from the first eigenvector, using 0 as threshold to differentiate both compartments and the gene density to label them.

TADs were identified using an in-house script that imported the "Chromosome" module of TADbit and added the raw and the ICE-normalized matrices of each chromosome separately. Filtered bins, due to low counts, were included in order to mask them when calling TADs. TAD insulation scores were obtained by first normalizing the different matrices for read depth in order for the scores to be comparable. Each matrix was then scaled to have 100M reads. Afterward, TAD insulation scores were obtained from the output given by the "hicFindTADs" program from HiCExplorer.

Compartment switching

BED files with a resolution of 50 Kbp were available from the compartments definition step. Each genomic bin of 50 Kbp had its corresponding compartment attributed. Pairwise comparisons between cell types -genome-wide and per-chromosome- were performed; the ratio of compartment switching was calculated as the number of genomic bins with a compartment change (A > B or B > A) divided by the total number of bins. From these files, a matrix file was created with 50 kbp-binned genomic coordinates as rows and cell types as columns, filled by the corresponding compartment labeling in each bin and cell type. Cell-specific A compartments were defined as those bins being compartment A in a cell type and compartment B in the remaining cell types.

3D-FISH and confocal microscopy

A total of 10 commercially available Bacterial Artificial Chromosomes (BACs) (Source BioScience) from mouse chromosomes 12 and 14 were selected according to their genomic location in A or B compartments (5 BACs for each compartment) (Table S4). The selected probes were separated by a range of genomic distances ranging from 66 kbp and 1.45 Mbp. Probes were selected based on their mappability and repetitive content in the mm10 genome assembly using the UCSC browser (https://genome.ucsc.edu/).

Probes were labeled with either dUTP-Dig (Sigma Aldrich) or dUTP-Cy3 (Enzo LifeSciences) by Nick Translation (Abbot Molecular). The 3D fluorescence *in situ* hybridization (3D-FISH) protocol was performed on mouse fibroblasts as following. Briefly, fibroblasts were cultured on slides overnight, whereas the germ cell suspension obtained after testis disaggregation was placed on slides

and incubated 1h at 37°C for cells to adhere on the surface. Slides were then fixed with 4% paraformaldehyde, washed in 1X PBS and then incubated in 60% Glycerol for 30-60 min, then finally snap-frozen in liquid nitrogen and kept in 50% formamide/2x SSC. As required, slides were thawed, treated with pepsin (0.01N HCl with 0.005% pepsin), washed with saline solutions (2x SSC, 50 mM $MgCl_2$, 1x PBS) and post-fixed with 1% paraformaldehyde. After 1h incubation in 50% formamide/2x SSC, the hybridization solution was placed on the slides. Both slides and probes were simultaneously denatured at 75°C for 2 min and then incubated for 48h at 37°C. Slides were washed in 2xSCC at 37°C and in 0.1x SSC at 60°C and dUTP-Dig was detected followed incubation with anti-Dig FITC (1:150) for 45 min at 37°C. Finally, slides were washed with 4x SSC with 0.2% Tween-20 and mounted with DAPI diluted in Vectashield. Germ cells from four males were subject to the protocol simultaneously.

Physical distances between hybridization signals were evaluated using a confocal microscopy (Leica SP5) using a 63x objective lens. Stacks of 0.34 μm-wide slices were captured for each cell nucleus, with a mean number of 20 stacks per nuclei. Images were reconstructed and analyzed using IMARIS (IMARIS Image Analysis Software), establishing nucleus volume (μm^3), physical distances between signals (μm) and relative physical distance between signals and nucleus surface (μm). For the data analysis, pairwise distances were computed for particular nuclei.

In total, we imaged 10 different loci (5 located in A compartments and 5 loci located in B compartment) across chromosomes 12 and 14. For each pair of probes, we measured the correlation between physical distances (in μm) and genomic distance (kbp) in pre-meiotic (fibroblasts and spermatogonia) and post-meiotic cells (round spermatids) (Figure S4; Table S4). A total of 527 measurements between seven pairs of probes were performed (Figure S6). We observed a positive correlation between physical and genomic distance (Spearman p value < 0.05) (Figure S4). But more importantly, we detected a differential pattern for both types of compartments (A and B) in round spermatids when compared to spermatogonia and fibroblasts (Figure S4). Lineal regressions were almost identical in spermatogonia and fibroblasts. In round spermatids, however, physical distances between pairs of probes were greater, confirming different chromatin organization in late spermatogenesis.

Western blot analysis of CTCF and cohesins in germ cells

Proteins from adult mice testis and fibroblast were extracted with RIPA buffer (NaCl 150 mM, Triton X-100 1%, Sodium deoxycholate 0.5%, SDS 0.1% and Tris-HCl 50 mM pH = 8.0). After protein quantification with the PierceTM BCA Protein Assay Kit (ThermoFisher Scientific), 6 replicates of both fibroblast and testis extract (three replicates of 30 μg and three of 40 μg protein) were denaturalized with 2x Laemmli and loaded into an 8% polyacrylamide gel until proteins reached the end of the gel [determined by the Precision Plus ProteinTM Dual Color Standards (Bio-Rad)]. Proteins were then transferred to a nitrocellulose membrane using the Trans-Blot® TurboTM Transfer System from Bio-Rad (10 minutes of transfer time). Next, membranes were stained with the Ponceau S solution (Sigma-Aldrich) to ensure that proteins had correctly transferred to the membrane. Then, the membranes were washed and incubated with blocking solution (TBS 1x with 0.1% Tween-20, 5% fat-free milk and 1x PBS) for at least an hour. Each set of testis and fibroblast protein extracts were incubated overnight at 4°C with anti-rabbit CTCF (1:2500), anti-rabbit RAD21L (1:2000) and anti-rabbit REC8 (1:2000) respectively, and all were simultaneously incubated with anti-rabbit ßTubulin (1:5000) as a control. Antibodies were detected the following day with 1-hour incubation with anti-rabbit HRP-PO (1:15000). Finally, membranes were detected with ClarityTM Western ECL Substrate and the results captured with the Molecular Imager VersadocTM (Bio-Rad).

Spermatocyte spreads and immunofluorescence analysis of CTCF and cohesins

Spermatocyte spreads were obtained from frozen mouse testis. Testes were mechanically disaggregated until obtaining a cell suspension in 1x PBS. The cell suspension was then distributed into different slides and incubated with 1% Lypsol for 16 minutes followed by a 20-minute incubation with 4% paraformaldehyde. Then slides were left to dry and washed with twice PhotoFlo 1% (Kodak) and then blocked with PBS-Tween-20 (0.005%). Slides were incubated overnight at 4°C with the following antibodies: anti-rabbit CTCF (1:50), anti-rabbit RAD21L (1:20) and anti-rabbit REC8 (1:20); one per slide and all of them combined with anti-mouse SYCP3 (1:400). Primary antibodies were detected with anti-rabbit Cy3 (1:200) combined with anti-mouse FITC (1:200). Slides were finally mounted with DAPI and analyzed with a fluorescence microscopy (Axiophot, Zeiss) coupled with a ProgRes ® CS10plus, Jenoptik camera. Representative images were captured with ACO XY (A. Coloma, Open Microscopy).

Stimulated emission depletion (STED) microscopy

Fab fragments were used for blocking and double labeling of REC8 and RAD21L antibodies (both raised in rabbit). STED microscopy (SP8, Leica) was used to generate the super-resolution images of REC8 and RAD21L foci along the chromosome axes. Secondary antibodies for STED imaging were conjugated to Alexa 555 and 488 (Invitrogen). Slides were mounted in Prolong Antifade Gold without DAPI. Fluorescence signals (red to green ratio) were measured along the 19 autosomal and XY axial elements of pachytene cells using the LAS X software from Leyca. Signal intensities were standardized and the overlay profiles of RAD21L and REC8 were plotted. Regression analysis was performed to determine the correlation between their profiles. The values of the coefficients of determination R^2 are shown in the scatterplots.

ChIP-sequencing

For chromatin immunoprecipitation, antibodies for CTCF (10 μl per sample) and cohesins RAD21L and REC8 (30 μl per sample) were used. Two biological replicates of ChIP-sequencing (ChIP-seq) were performed using FACS-sorted primary spermatocytes at P/D

stage (14 million of cells) and round spermatids (20 million of cells) from adult mice. In short, cells were incubated on ice in lysis buffer I (5 mM PIPES, 85 mM KCl, 0.5% NP-40, Protease Inhibitors) and lysis buffer II (1% SDS, 10 mM EDTA, 50 mM Tris-HCl, Protease Inhibitors). Samples were then sonicated with the Biorruptor pico (30 s ON, 30 s OFF, 10 cycles) to obtain fragments around 200 bp. Sonicated lysates were centrifuged, and the supernatant was diluted in cold IP buffer (Diagenode). A small aliquot of each sample was kept as input and the remaining was divided in three aliquots, where antibodies for CTCF, RAD21L and REC8 were added (one in each aliquot) and then incubated overnight at 4°C. The antibody-chromatin pull-down was performed with Unblocked Protein A beads (Diagenode) and beads were then eluded with elution buffer (1% SDS, 0.1M $NaHCO_3$). After elution samples were centrifuged, and the supernatant was incubated with 200 mM NaCl at 65°C overnight to reverse the crosslink. Finally, proteins were digested and the DNA purified with phenol:chloroform:isoamyl alcohol. Libraries were prepared for each sample, repairing fragment ends by incubating the samples for 30 minutes with the NEBNext end repair mix (New England Biolabs). Then samples were purified with AMPure beads. Samples were then incubated with the A-tailing NEBNext mix (New England Biolabs) and purified again with AMPure beads. Then, adaptors were ligated to the sample, which was subsequently purified with AMPure beads (0.8X). Libraries were PCR-enriched using NEBNext indexed primers, 12 PCR cycles for RAD21L and CTCF samples and 14 cycles for REC8 samples. Libraries were finally purified with AMpure beads (0.7X).

ChIP-seq peak calling and annotation

Quality check and trimming were performed using BBDuk. Setting a minimum read length of 35 bp and a minimum Phred quality score of 20, adapters and low-quality reads were removed while preserving their longest high-quality regions. Single end reads were obtained for CTCF ChIP and paired end reads for the cohesin ChIP. Reads were trimmed and mapped to the mouse reference genome (mm10) using a Galaxy server. After mapping, non-unique mapped reads with a mapping quality less than 30 were filtered out with SAMtools (Li et al., 2009). Then, to assess read coverage distribution across the genome, bigWig files for each sample were generated with DeepTools (Ramírez et al., 2016). Following the server recommendations, filtered files were merged with Picard (Broad Institute) and split again with SAMtools before peak calling. Peak calling was performed using MACS2 (Feng et al., 2012). To find the optimal parameters for running the peak call function, a cross-correlation analysis between reads mapping to plus and minus strands was performed, and the d parameter was estimated for each sample. Peaks were called using as extension size the d estimated for each sample respectively. Coverage, reads and peaks were visualized with IGVtools (Thorvaldsdóttir et al., 2013).

BED peak files were imported to Rstudio and annotated to the reference mouse genome (mm10) using the R/bioconductor package ChIPseeker (Yu et al., 2015) together with the knownGene table from the UCSC resources (TxDb.Mmusculus.UCSC.mm10.knownGene). The number of peaks overlapping between CTCF and cohesins and between cell types was obtained by using the bioconductor package ChIPpeakAnno (Zhu et al., 2010). ChIP-seq data was represented using the packages ggplot2 (Wickham, 2016) and karyploteR (Gel and Serra, 2017) in combination with ChIPseeker and ChIPpeakAnno.

RNA-sequencing

Full-length single-cell RNA sequencing libraries were prepared using the Smart-seq2 protocol (Picelli et al., 2014) with minor modifications. Pools containing between 20,000 and 40,000 cells were obtained by FACS-sorting for four cell types (spermatogonia, primary spermatocytes at P/D, round spermatids and sperm) from adult mice. Four independent biological replicated were included in the analysis. Briefly, cells were sorted into 1.5ml eppendorfs containing lysis buffer. Reverse transcription was performed using SuperScrpit II (Invitrogen) in the presence of oligo-dT30VN, template-switching oligonucleotides and betaine. The cDNA was amplified using the KAPA Hifi Hotstart ReadyMix (Kappa Biosystems), ISPCR primer and 16 cycles of amplification. Following purification with Agencourt Ampure XP beads (Beckmann Coulter), product size distribution and quantity were assessed on a Bioanalyzer using a High Sensitvity DNA Kit (Agilent Technologies). Amplified cDNA was fragmented using Nextera® XT (Illumina) and amplified with indexed Nextera® PCR primers. Products were purified twice with Agencourt Ampure XP beads and quantified again using a Bioanalyzer High Sensitivity DNA Kit. Sequencing of Nextera® libraries was carried out on a HSeq2500 (Illumina) to obtain > 30 million pair ends reads per sample.

RNA profiling

The Artificial Intelligence RNA-seq Software as a Service (SaaS) platform (https://transcriptomics.cloud) was used to analyze RNA-seq data. AIR accepts raw next generation sequencing Illumina FastQ data as input. RNA-seq data was uploaded to the site and validated in order to automatically pair forward and reverse files (in case of paired-end samples) as well as to check its format and integrity. Quality was assessed using FastQC. A new analysis was defined from the "new analysis" screen, where the samples to be included were selected for analysis, along the reference genome (> 120,000 available genomes from NCBI, Ensembl and JGI). The analysis included quality trimming, Differential Gene Expression (DGE) followed by a Gene Ontology Enrichment Analysis (GOEA). Once the analysis was launched, bad quality reads were removed using BBDuk by setting a minimum length of 35 bp and a minimum Phred-quality score of 25. Afterward, high quality reads were mapped against the reference genome with STAR (Dobin et al., 2013) using the end-to-end alignment mode and gene expression quantification was performed with featureCounts (Liao et al., 2014).

The statistical analysis started by filtering lowly expressed genes using HTSFilter (Rau et al., 2013). Four statistical methods were then used for the identification of differentially expressed genes: DESeq2 (Love et al., 2014), edgeR (Robinson et al., 2010), EBSeq (Leng et al., 2013) and NOISeq (Tarazona et al., 2011). Data normalization was performed with the Trimmed Mean of M-values (TMM) method. Finally, GOEA was performed with in-house scripts based on hypergeometric tests (Tian et al., 2017). Multiple testing corrections controlling false positives from high-throughput experiments were also performed with the Benjamini-Hochberg method. The statistics section included: the Principal Component Analysis (PCA) clusterization of the samples, general plots for the interpretation of the experiment and several tabs in which the Differential Expressed Genes (DEG) and GOEA data is shown in different tables.

Specifically, we selected the mouse genome (GRCm38) from the Ensembl release 89. NOISeq was used to explore the DEG due to the variability of biological replicates. Raw expression and FPKM (Fragments Per Kilobase Million) values were downloaded from AIR. The Principal Component Analysis (PCA) was performed using the noise correction function from the NOISeq package. The raw expression values were converted to Counts Per Million (CPM) using edgeR and, afterward, expression values for each cell type were averaged for further analyses.

Analysis of correspondence between compartments and gene expression

Cell-specific A-B compartments were intersected with BEDTools (version 2.26) against a BED file with the TSS of genes derived from the GRCm38 gene annotation from Ensembl (release 89). Genes in each compartment were grepped (Bash command) with the table of FPKM values downloaded from AIR (see above), producing the expression profiles represented as boxplots for each cell type and compartment. Statistical significance among pairwise comparisons was tested using the Wilcoxon test.

For GOEA in cell-specific A-compartments we extracted gene Ontology (GO) terms from expressed genes (CPM > 1) located in A compartments. GOEA was performed following the approach used by AIR on the DEG. It consists of a series of hypergeometric tests carried out for each GO term thus identifying significant enriched GO terms relative to the expected genome background. P values were corrected with the Benjamini-Hochberg procedure to reduce false positives. We considered as significantly enriched the terms with corrected P values $\leq$ 0.01. Results were displayed using bubble plots.

Moreover, expressed genes with peaks on their promoters were classified according to cell type and protein (CTCF, RAD21L and REC8). Genes were then analyzed with PANTHER (Mi et al., 2017) using the online tool AmiGO (Carbon et al., 2008).

Local insulation, cohesin occupancy and gene expression

The GRCm38 gene annotation from Ensembl (release 89) was downloaded in GTF format. It was parsed with an in-house script to extract in BED format the promoter regions 2 kbp upstream the Transcriptional Start Site (TSS) for each gene. Afterward, ChIP-seq peaks were intersected with BEDTools to obtain gene lists with ChIP-seq peaks in their promoters. These gene lists were grepped (Bash command) with the downloaded table with the expression values from the AIR platform (see below).

Protein peaks were associated with changes in the corresponding TAD insulation score or gene expression using the function meanInRegions of the R package regioneR (version 1.10) (Gel et al., 2016). This package generates an expected distribution from 10.000 permutations from the values (e.g., TAD insulation score or FPKM values) observed in random genomic locations, thus calculating the Z-score of the TAD insulation score or the gene expression observed on ChIP peaks. Z-scores from flanking regions (+/− 250 Kbp) of peaks were calculated with the function localZScore.

Meta-border plots were created using ICE-normalized matrices normalsed by the number interactions expected at a given distance with "hicFindEnrichedContacts" program from the HiCExplorer package (parameters: "–method obs/exp," "–perchr"). Subsequently, sub-matrices of interaction counts 5 Mbp up and downstream of specific regions (e.g., TAD boundaries or protein peaks) were generated and averaged among them. The interaction counts underwent a log10 transformation and were plot with "hicPlotTADs" program from HiCExplorer.

X chromosome evolutionary strata and ampliconic regions

Mouse X strata were extrapolated from the human X chromosome using synteny information (https://www.ensembl.org/Mus_musculus/Location/Synteny?r=X). Likewise, the boundaries of each amplicon in the current assembly were defined by flanking paralogs (Table S7).

QUANTIFICATION AND STATISTICAL ANALYSIS

All sequencing data was checked and trimmed with BBDuk (Bushnell, 2014). Hi-C data was processed with TADbit (version 0.2.0.23) to obtain raw interaction matrices, compartments and TADs. HiCExplorer was used to: (i) normalize raw interaction matrices, (ii) create interaction heatmaps, (iii) predict averaged contact probabilities P(s) and (iv) obtain TAD insulation scores. Correlation values between biological replicates were performed using HiCRep (Yang et al., 2017). Normalized interaction matrices were exported with HiCExplorer to the GInteractions format and imported in R to calculate the inter-intra-chromosome interaction ratio. Normalized interaction matrices were scaled with a factor of 1,000,000/sum(matrix) with a custom Python script, exported with HiCExplorer to GInteractions format and imported in R to create boxplots. Differences between cell types were assessed with the Wilcoxon test (p value < 0.05).

ChIP-seq data was processed using the Galaxy server. BigWig files for each sample were generated with DeepTools (Ramírez et al., 2016) and peaks were called using MACS2 (Feng et al., 2012). BED peak files were imported to R and annotated using the R/Bioconductor package ChIPseeker (Yu et al., 2015) and UCSC resources (TxDb.Mmusculus.UCSC.mm10.knownGene). ChIPpeakAnno (Zhu et al., 2010) was used for peak intersection. Changes in the insulation score on peaks were statistically assessed using the function meanInRegions of the R package regioneR (version 1.10) (Gel et al., 2016).

RNA-seq data was processed with the Artificial Intelligence RNA-seq (AIR) Software as a Service (SaaS) platform (https://transcriptomics.cloud). In this case, raw expression values were converted to Counts Per Million (CPM) using edgeR, and averaged for each cell type. FPKM values were averaged for each cell type and BEDTools (version 2.26) was used to intersect genomic coordinates to relate gene expression with compartments, insulation scores and ChIP-seq marks. Differences in gene expression in the intersected regions between cell types were assessed by means of Wilcoxon tests (p value < 0.05).

DATA AND CODE AVAILABILITY

The accession number for the Hi-C, RNA-seq and ChIP-seq data reported in this paper is GEO: GSE132054.

Cell Reports, Volume *28*

Supplemental Information

Three-Dimensional Genomic Structure and Cohesin Occupancy Correlate with Transcriptional Activity during Spermatogenesis

Covadonga Vara, Andreu Paytuví-Gallart, Yasmina Cuartero, François Le Dily, Francisca Garcia, Judit Salvà-Castro, Laura Gómez-H, Eva Julià, Catia Moutinho, Riccardo Aiese Cigliano, Walter Sanseverino, Oscar Fornas, Alberto M. Pendás, Holger Heyn, Paul D. Waters, Marc A. Marti-Renom, and Aurora Ruiz-Herrera

Figure S1

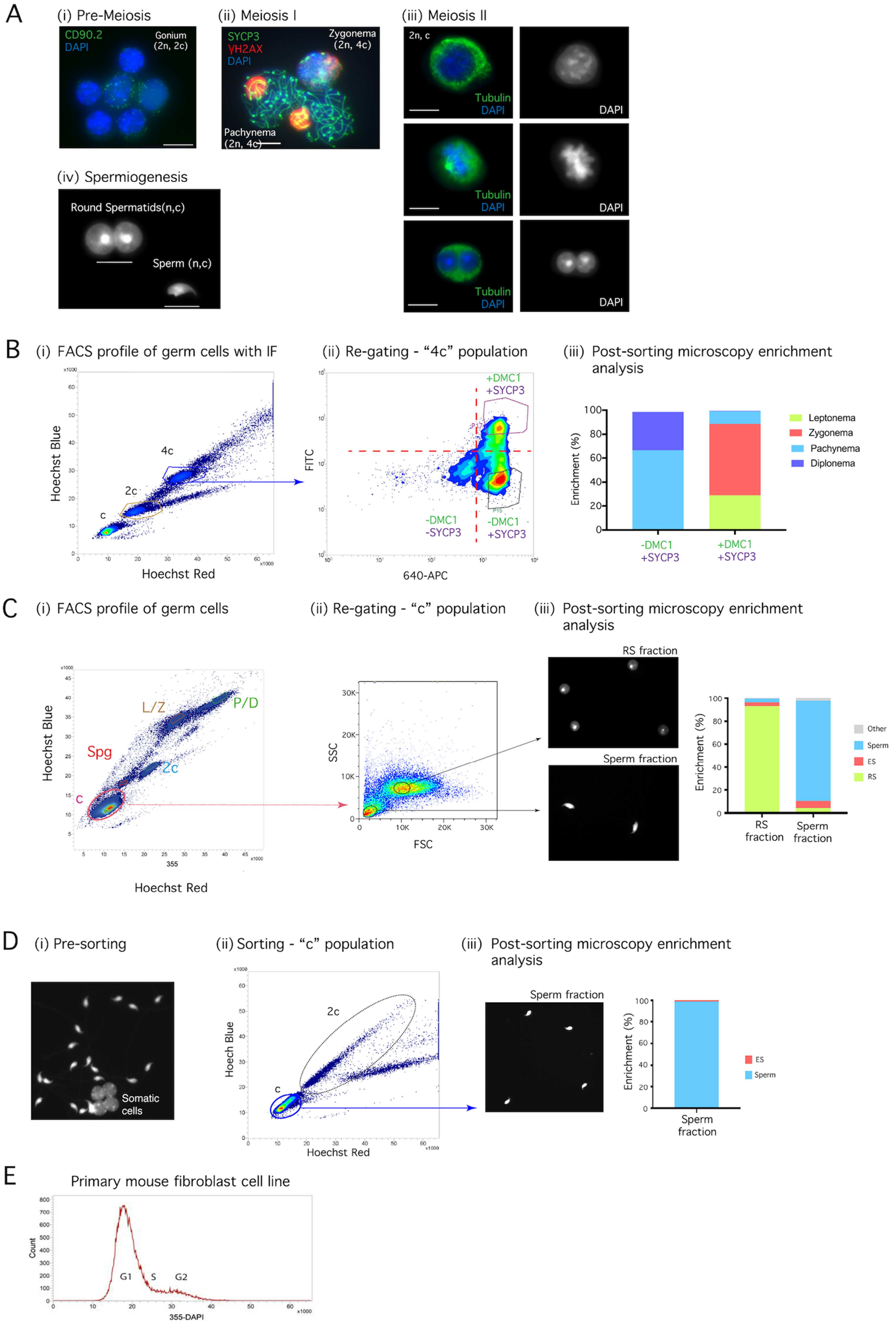
A
(i) Pre-Meiosis
CD90.2
DAPI
Gonium (2n, 2c)
(ii) Meiosis I
SYCP3
γH2AX
DAPI
Zygonema (2n, 4c)
Pachynema (2n, 4c)
(iii) Meiosis II
2n, c
Tubulin
DAPI
(iv) Spermiogenesis
Round Spermatids(n,c)
Sperm (n,c)
B
(i) FACS profile of germ cells with IF
(ii) Re-gating - "4c" population
(iii) Post-sorting microscopy enrichment analysis
Hoechst Blue
Hoechst Red
4c
2c
c
FITC
640-APC
+DMC1 +SYCP3
-DMC1 -SYCP3
-DMC1 +SYCP3
Enrichment (%)
Leptonema
Zygonema
Pachynema
Diplonema
C
(i) FACS profile of germ cells
(ii) Re-gating - "c" population
(iii) Post-sorting microscopy enrichment analysis
L/Z
P/D
Spg
2c
c
SSC
FSC
RS fraction
Sperm fraction
Other
Sperm
ES
RS
D
(i) Pre-sorting
(ii) Sorting - "c" population
(iii) Post-sorting microscopy enrichment analysis
Somatic cells
Hoech Blue
Hoechst Red
E
Primary mouse fibroblast cell line
Count
G1
S
G2
355-DAPI

Figure S1: FACS strategies for isolation of highly enriched germ cell populations, related to Figure 1. (A) Representative immunofluorescence images for the flow-sorted germ cells populations. (i) Pre-meiotic cells include spermatogonia positive for CD90.2 (green). (ii) Meiosis I includes spermatocytes I at leptonema/zygonema and pachynema/diplonema stages. Synaptonemal complexes (SYCP3) are labelled in green, γH2AX in red and DNA in blue (DAPI). (iii) Meiotic II cells represent a heterogeneous population including spermatocytes II in G_1, metaphase and diakinesis. Tubulin is labelled in green and DNA in blue (DAPI). (iv) The spermiogenesis stage includes round spermatids and sperm. Legend: n, chromosome number per cell; c, number of chromatids per chromosome. Scale bar: 10 μm. (B) Highly enriched sub-populations of primary spermatocytes. (i) Flow cytometry Hoechst Blue (UV355-460/50) and Hoechst Red (UV355-670/30) plot after "in solution" immunofluorescence with DMC1 and SYCP3. Germ cells are differentiated by distinct DNA content: 4c, 2c and c. (ii) Re-gating of the 4c population displaying three different cell populations: +DMC1/+SYCP3, -DMC1/+SYCP3 and -DMC1/-SYCP3. (iii) Enrichment values for +DMC1/+SYCP3 and -DMC1/+SYCP3 sorted populations. (C) Isolation of highly enriched round spermatids and sperm through re-gating. (i) Main FACS profile from whole testis, where primary gating is established for each germline population. (ii) Isolation of round spermatids and sperm, where cells are re-gated based on how cells project a side shadow (side scatter, SSC) and a forward shadow (forward scatter, FSC), attaining highly enriched fractions of these populations. (iii) Representative images and enrichment values for round spermatids (RS) and sperm fractions after re-gating. Legend: RS – round spermatids, ES – elongated spermatids. (D) Isolation of sperm from caudate epididymis. (i) Representative cells obtained from the caudate epididymis stained with DAPI prior to FACS. (ii) Flow cytometry Hoechst Blue (UV355-460/50) and Hoechst Red (UV355-670/30) plot showing isolated sperm from caudate epididymis. Two populations of cells are evidenced (2c and c). Re-gating of the c fraction resulted in isolated sperm. (iii) Representative image and enrichment values after sorting. Legend: ES – elongated spermatids. (E) FACS profile of mouse primary fibroblast cell culture.

Figure S2

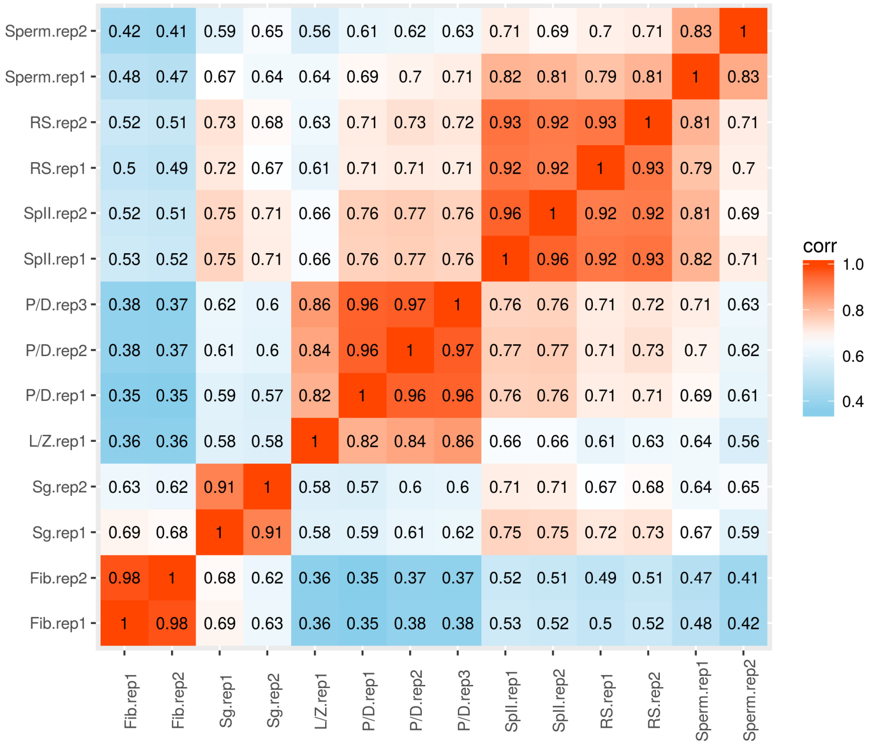

Figure S2: Correlation among Hi-C replicates, related to Figure 2. Heatmap showing the correlation values among replicates based on the pairwise similarity score calculated using HiCRep. Legend: Fib – Fibroblast, Sg – Spermatogonia, L/Z – Leptonema/Zygonema, P/D – Pachynema/Diplonema, SpII – Spermatocytes II, RS – Round Spermatids, Rep#: replicates.

Figure S3

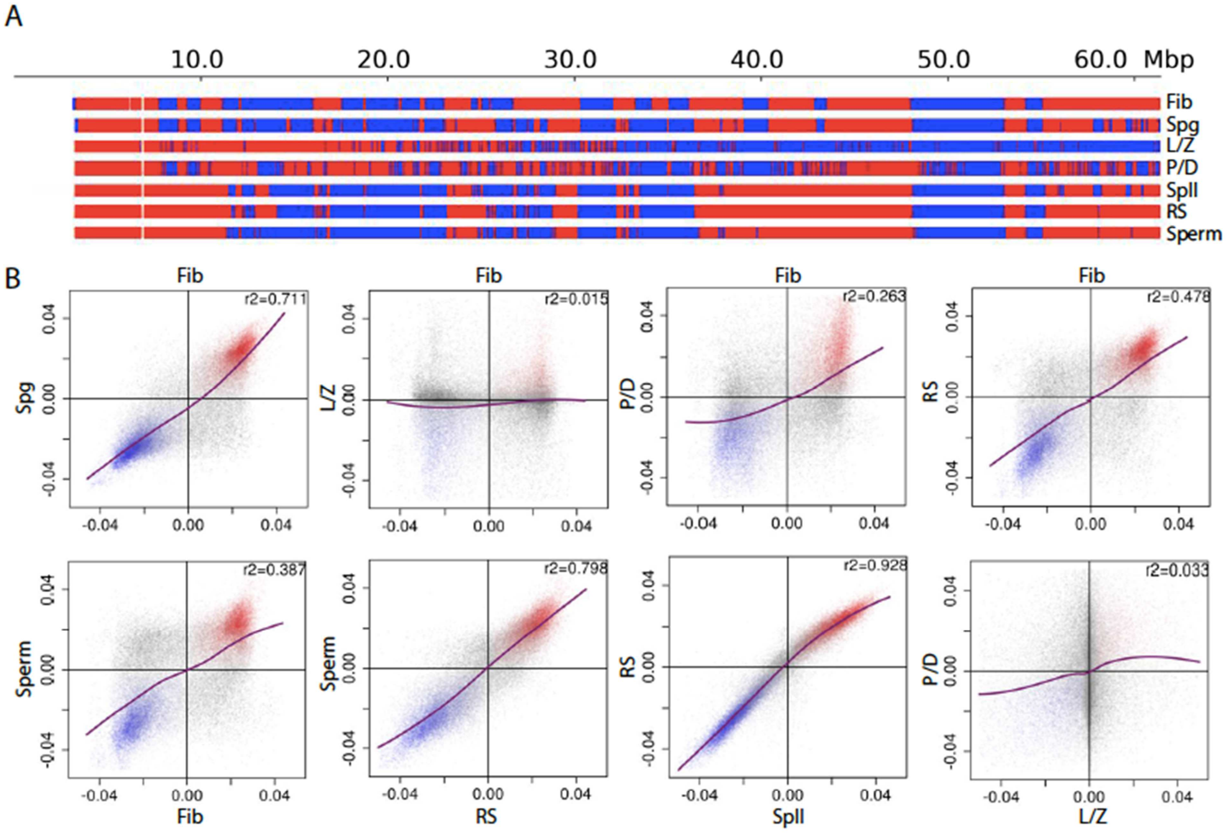

Figure S3: Compartment switching during spermatogenesis, related to Figure 3. (A) Representation of the genomic regions corresponding to A (red) and B (blue) compartments in mouse chromosome 19 for all cell types analysed. The distribution of gene density is also displayed at the bottom of the figure (purple spikes). (B) Pair-wise representation of eigenvectors between cell types genome-wide. Each dot represents a 50Kbp bin in the genome. Bins representing A compartment conservation are depicted in red, whereas in blue are depicted bins with B compartment conservation. Bins with unclear signal or compartment switching are represented in grey. The purple line is a LOESS curve showing the tendency of the compartment switching. Correlation values are represented for each pair-wise comparison. Legend: Fib – Fibroblast, Spg – Spermatogonia, L/Z – Leptonema/Zygonema, P/D – Pachynema/Diplonema, SpII – Spermatocytes II, RS – Round Spermatids.

Figure S4

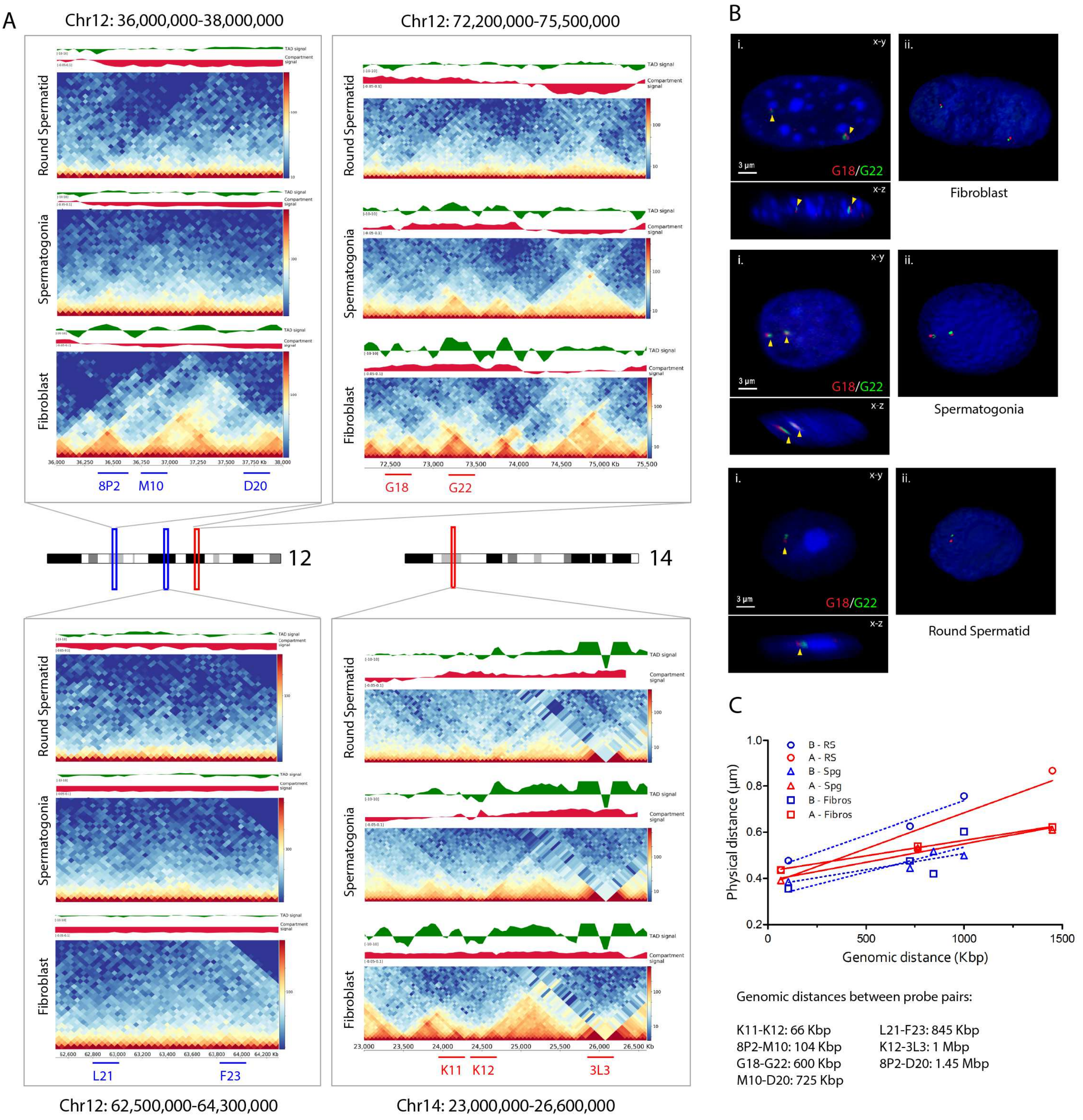

Figure S4: Analysis of A/B compartments by 3D-FISH, related to Figure 3. (A) Heatmaps from the four chromosomal regions analysed by 3D-FISH in mouse chromosomes 12 and 14. Probes located at A-compartments are red, whereas probes located at B compartments are blue. (B) Two-colour 3D-FISH images of example pairs of probes within A-compartments in fibroblasts, spermatogonia and round spermatids (see supplementary methods for probe localisation). In all cases, left panels (i) represent the confocal x-y (upper section) and x-z sections (lower section), whereas right panels (ii) represent IMARIS three-dimensional reconstructions. (C) Lineal plot of the median genomic distances (in Kbp) between pairs of probes versus physical distances (in mm) in fibroblasts (n = 275), spermatogonia (n = 155) and round spermatids (n = 97) for both types of compartments (A in red and B in blue).

Figure S5

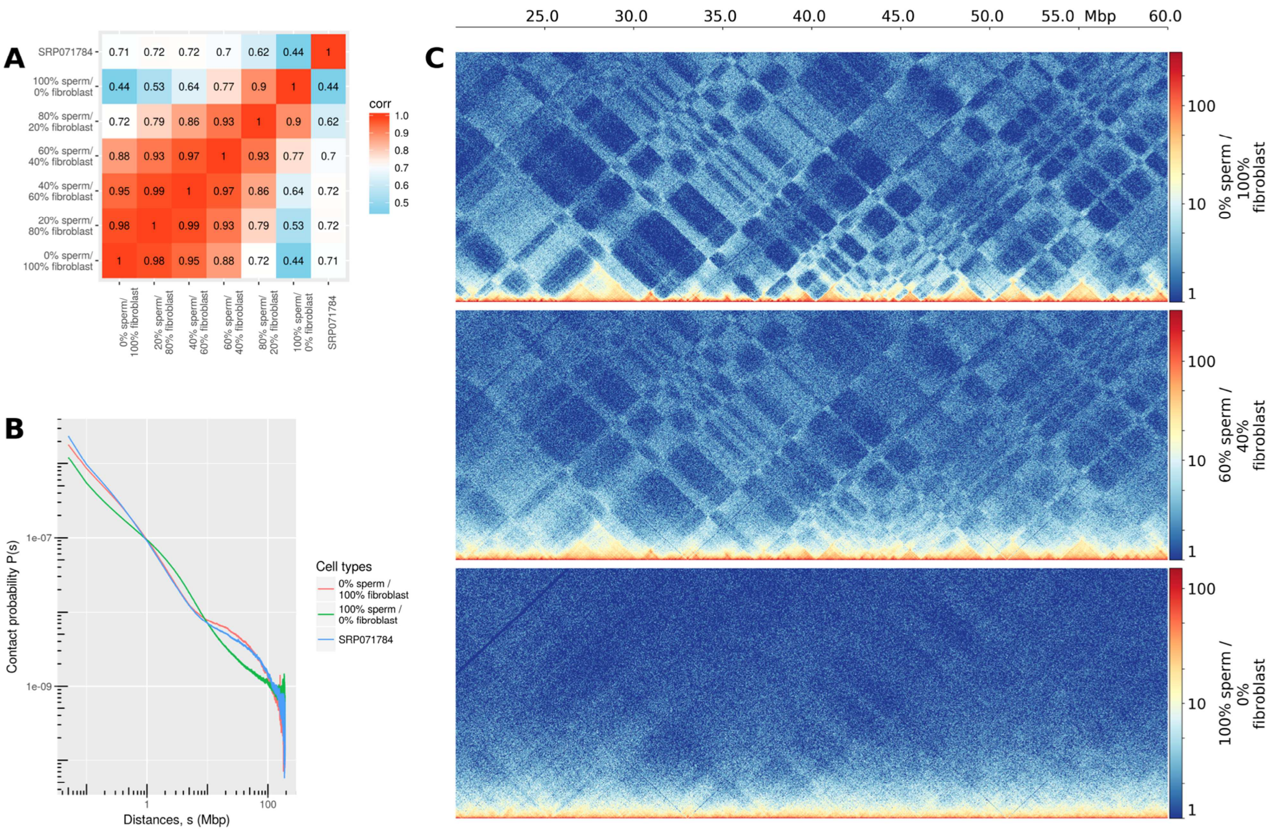

Figure S5: Simulations of samples with different fibroblast and sperm content, related to Figure 3. (A) Heatmap showing correlation values, based on the pairwise similarity score calculated using HiCRep, among samples with different fibroblast and sperm content as well as the merged sperm replicates from SRP071784 (retrieved from Jung et al. 2017). (B) Contact probability as a function of genomic distance for samples with different fibroblast and sperm content as well as the merged sperm replicates from SRP071784. (C) Chromosome 8 region-specific ICE-corrected heatmaps at 50 kbp for samples with different fibroblast and sperm content, as well as from SRP071784.

Figure S6

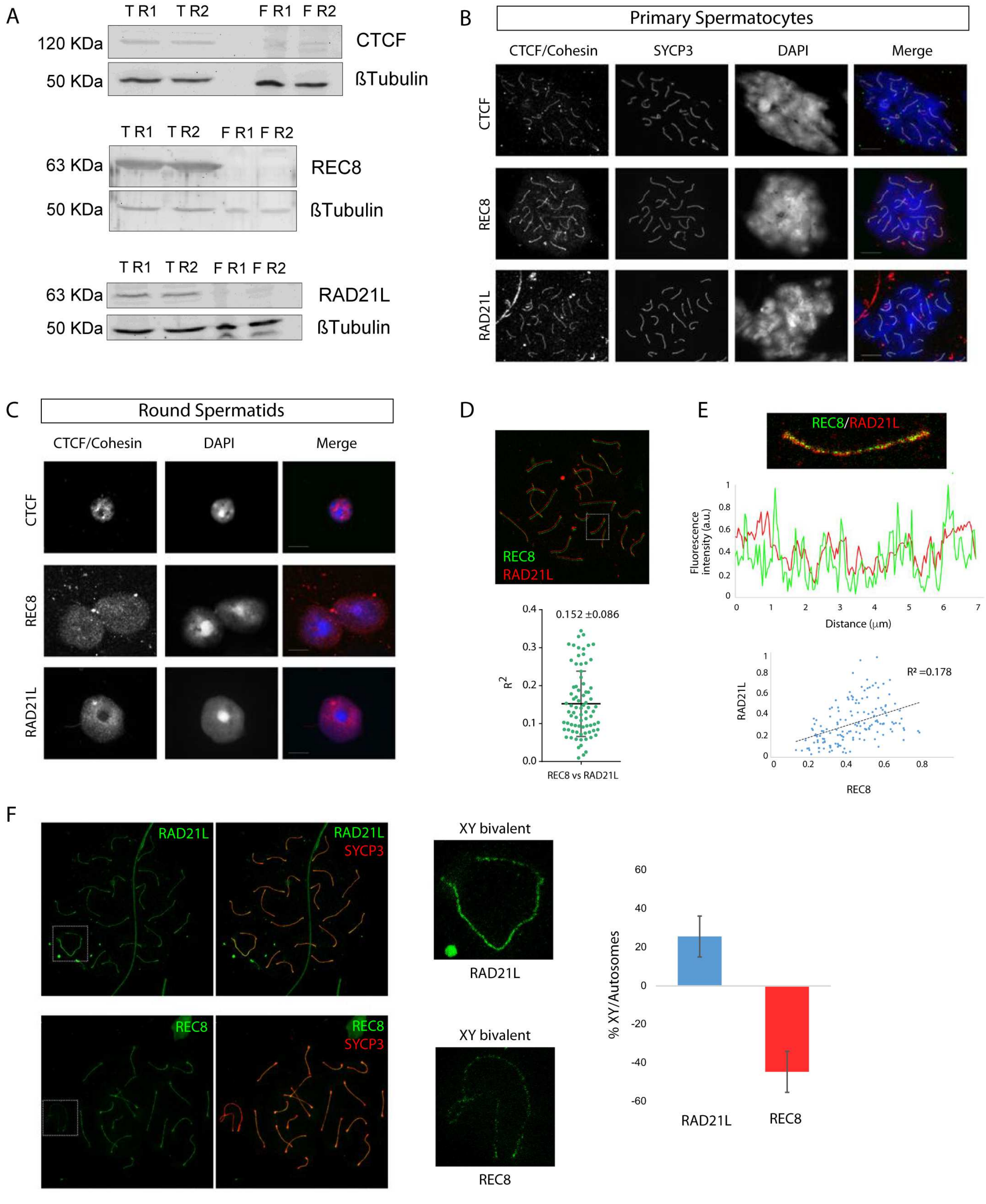

Figure S6: Expression and intracellular localisation of CTCF and cohesins (REC8 and RAD21L) in mouse germ cells, related to Figures 5 and 6. (A) Western blotting analyses on whole nuclear extracts from mouse testis (two replicates) and fibroblasts (two replicates). Abbreviations: T– testis, F –fibroblast, R# – replicate. The band corresponding to CTCF was detected at 120 KDa, REC8 and RAD21L at 63 KDa, approximately. The β-tubulin loading control was detected at about 50 kDa. (B) Examples of immunofluorescence images showing the intracellular distribution of CTCF, RAD21L and REC8 in primary spermatocytes at pachytene stage. Double immunolabelling of CTCF (red)/ RAD21L (red)/ REC8 (red) with SYCP3 (green) are shown. Scale bar: 10 μm. (C) Examples of immunofluorescence images showing the intracellular distribution of CTCF, RAD21L and REC8 in round spermatids.

Double immunolabelling of CTCF (red)/ RAD21L (red)/ REC8 (red) with SYCP3 (green) are shown. Scale bar: 10 μm. (D) Double immunolabelling of REC8 (green) and RAD21L (red) at pachytene stage showing only partial overlapping at the chromosome axes by STED microscopy. Plot of the mean correlation between REC8 and RAD21L at the autosomes (n=76 axial elements, AEs; mean ± s.d.). (E) Example of an autosome axes immunolabelled for REC8 (green) and RAD21L (red) (upper panel). Plot represents normalised signal intensity profile of REC8 (green) with RAD21L (red) (middle panel). Lower plot shows regression analysis of the correlation between REC8 and RAD21L (lower panel). (F) Example of differential immunolabelling of (i) RAD21L (increased labelling) and (ii) REC8 (decreased labelling) at the XY bivalent in a spermatocyte at pachytene. (iii) Magnification of the XY bivalent is shown for each cohesin. (iv) Plot representation of the labelling gain/loss of RAD21L and REC8 fluorescence intensity (depicted in %) along the XY axes in comparison with autosomes (n=19 cells).

Figure S7

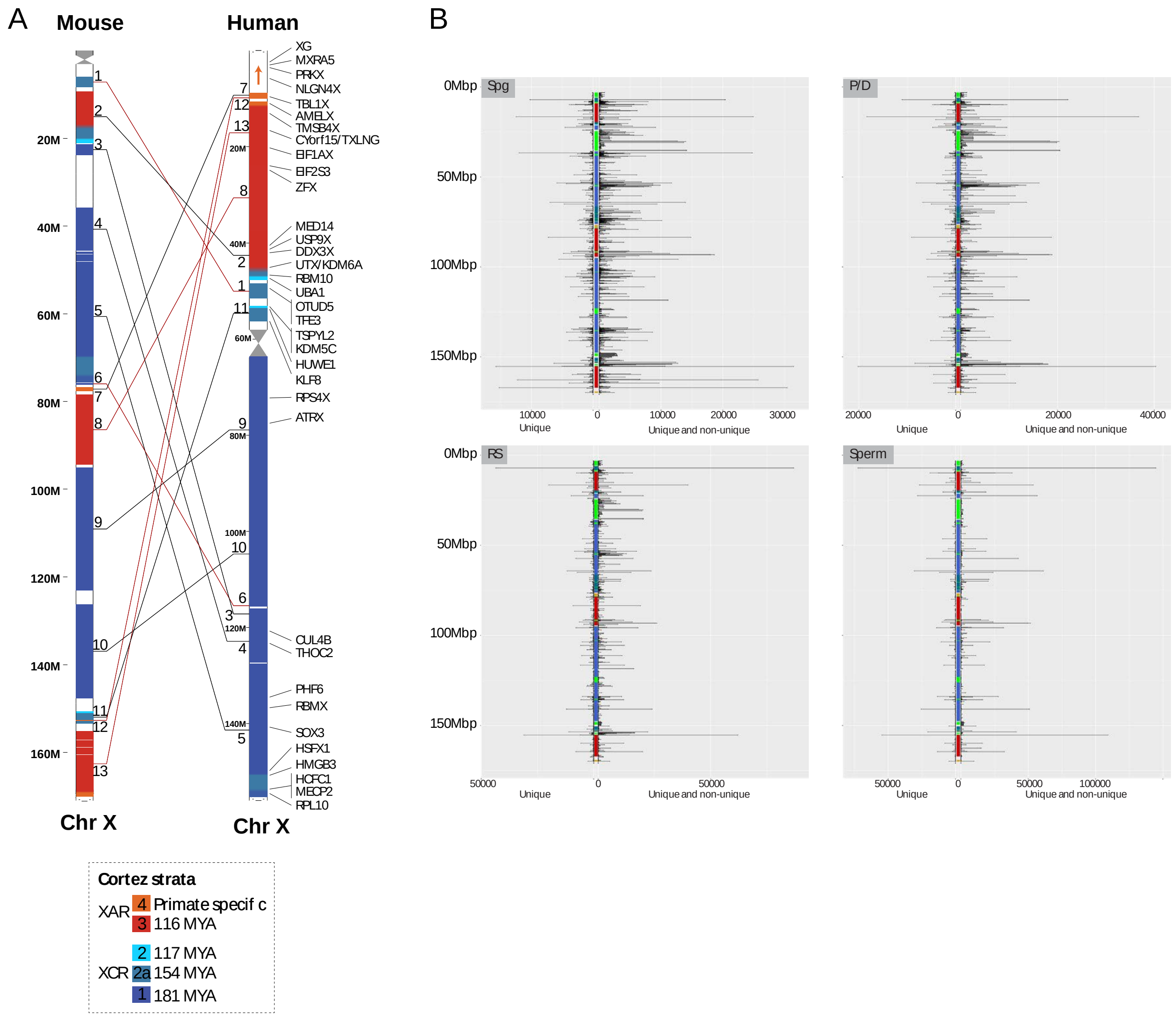

Figure S7: Evolutionary strata and ampliconic regions in the mouse X chromosome, related to Figure 7. (A) Evolutionary strata (from Cortez et al., 2014) for the human and mouse X chromosomes. Strata were assigned to the mouse X using human synteny (https://ensembl.org/Homo_sapiens/Location/Synteny?r=X). White regions are breaks in synteny that usually correspond to amplicons (see panel B). (B) RNA-seq tag counts on the X chromosome in spermatogonia (Spg), pachynema/diplonema (P/D), round spermatids (RS) and sperm. Colours in the centre represent evolutionary strata (as in panel A), green represent amplicons (see Table S7 for coordinates). Peaks to the left represent counts for uniquely mapped RNA-seq reads. Peaks to the right are counts for both uniquely mapped and multi-mapped RNA-seq reads. As expected, in the ampliconic regions counts are higher when including multi-mapped reads.
